# Supplementary material for: Biosensor for branched-chain amino acid metabolism in yeast and applications in isobutanol and isopentanol production
Source: Nat Commun. 2022 Jan 12;13:270. doi: 10.1038/s41467-021-27852-x (PMC8755756; doi:10.1038/s41467-021-27852-x)
Supplement: Supplementary file 1 — Supplementary Information [file 41467_2021_27852_MOESM1_ESM.pdf]

**Supplementary Information for:**

# Biosensor for branched-chain amino acid metabolism in yeast and applications in isobutanol and isopentanol production

Yanfei Zhang [1], Jeremy D. Cortez [# 2], Sarah K. Hammer [# 1], César Carrasco-López [1], Sergio Á. García Echauri [1], Jessica B. Wiggins [3], Wei Wang [3] and José L. Avalos * [1, 2, 4, 5]

[1] Department of Chemical and Biological Engineering; [2] Department of Molecular Biology; [3] Genomics Core Facility, Lewis-Sigler Institute for Integrative Genomics; [4] Andlinger Center for Energy and the Environment, [5] High Meadows Environmental Institute, Princeton University, Princeton, NJ

# These authors made equal contribution

*Corresponding author: José L. Avalos

Department of Chemical and Biological Engineering,

Princeton University,

101 Hoyt Laboratory, William Street, Princeton, NJ 08544, USA

Phone office: +1 (609) 258-9881

Phone lab: +1 (609) 258-0542

Fax: +1 (609) 258-1247

Email: javalos@princeton.edu

**Supplementary Notes:**

**Supplementary Note 1: Additional details on the development and evaluation of different biosensor constructs.** The BCHA biosensor is based on the transcriptional regulator Leu3p which activates genes involved in BCAA biosynthesis when bound to α-IPM and represses them when unbound[1,2]. Because the *LEU1* promoter ($P_{LEU1}$) is regulated by Leu3p, a yEGFP reporter downstream of $P_{LEU1}$ can be used to monitor α-IPM intracellular levels, and thus BCAA biosynthetic activity. The enzyme responsible for α-IPM synthesis (Leu4p), however, is feedback inhibited by leucine which would block the biosynthetic pathway and thus the effector metabolite of the biosensor. Therefore, we tested three different *LEU4* mutants insensitive to leucine inhibition (*LEU4*$^{\Delta S547}$, *LEU4*$^{1\text{-}410}$, and *LEU4*$^{1\text{-}474}$), plus the wild type as control, expressed constitutively using the TPI1 promoter ($P_{TPI1}$). Because Leu4p is active as a dimer, it is possible that heterodimers of the endogenous wild type copy and mutant variants are formed ($Leu4^{WT}/Leu4^{variant}$), explaining some of the difference in performance we observed. When the BCHA biosynthesis pathway (plasmid pJA182) is overexpressed in *leu2*Δ strains harboring different biosensor constructs, the *LEU4*$^{1\text{-}410}$ variant lacking the leucine regulatory domain, produces the largest increase in fluorescence especially when a PEST tag is fused to yEGFP (Supplementary Fig. 2a). While *LEU2* deletion favors isobutanol production, the pathway bottleneck introduced by this deletion increases the accumulation of α-IPM, which explains why constructs with stable (untagged) yEGFP have higher background than those with a PEST tag and why destabilizing yEGFP helps lower the background and increase the apparent dynamic range (Fig. 1a, Supplementary Fig. 2a). The reason we chose to delete *LEU2* and not *LEU1* is twofold. First, the parent strain already contains a *LEU2* deletion as an auxotrophic marker so it is not necessary to also delete *LEU1* to prevent flux towards leucine biosynthesis. Additionally, we

hypothesize that the reversible activity of Leu1p could help reduce the biosensor background by dampening the accumulation of α-IPM. *LEU2* deletion also causes leucine auxotrophy, requiring supplementation of this amino acid in the growth medium; however the *LEU4*$^{1\text{-}410}$ leucine-insensitive mutant keeps the biosensor operational.

In contrast, for isopentanol production it is necessary to express *LEU2* (Supplementary Fig. 1). Therefore, to obtain a biosensor configuration suitable for isopentanol sensing, we tested the same constructs above but in a *LEU2* strain (Supplementary Fig. 2b). Because α-IPM is an intermediate metabolite of isopentanol biosynthesis (Supplementary Fig. 1), intracellular concentrations of α-IPM are expected to be substantially lower in strains engineered to make isopentanol than in *leu2Δ* strains that produce isobutanol, in which α-IPM can be considered to be a byproduct (Supplementary Fig. 1). Thus, using a PEST-tagged yEGFP in *LEU2* strains is counterproductive, and an untagged yEGFP enhances the biosensor sensitivity and apparent dynamic range (Supplementary Fig. 2b). In addition, we found that deleting the endogenous genes encoding for α-IPM synthases (*LEU4* and *LEU9*) reduces the biosensor background in *LEU2* strains. However, this requires replacing Leu4$^{1\text{-}410}$, which depends on heterodimerization with endogenous *LEU4* for activity, with another leucine-insensitive Leu4p mutant. The Leu4p harboring a Ser547 deletion (Leu4$^{\Delta S547}$)[3] is a leucine-insensitive mutant that is active as homodimers in the absence of wild type *LEU4*[4] *,* which in *LEU2* strains gives the largest apparent dynamic range (Supplementary Fig. 2b).

It is important to note that previous studies[5-8] have shown that small protein fusions cause no measurable effect on GFP quantum yield or brightness, including the PEST-Cln2 tag used in this study[5], which does not seem to impede biosensor functionality.

**Supplementary Note 2: Biosensor dynamic range estimation.**

Because the biosensor is based on the ability of Leu3p to respond to intracellular levels of α-IPM, it is difficult to measure its dynamic range. Feeding α-IPM or α-KIV in the media at relatively low concentrations (< 80 μM) helps confirm the biosensor response to elevated levels of α-IPM and its precursor (Supplementary Fig. 3a,b). However, the biosensor response saturates when feeding α-IPM above ~80 μM, or α-KIV above ~300 μM (Supplementary Fig. 3c,d). Since the stoichiometric ratio between these metabolites in the biosynthetic pathway is one to one, and the biosensor response is linear with respect to both metabolites up to ~ 80 μM, the saturation at different concentrations suggests there may be differences in the rates of cellular uptake of these metabolites, which likely become limiting at different concentrations. Similarly, differences in their metabolic conversion rates could greatly affect the biosensor response when feeding metabolites to the media. Therefore, caution should be taken when interpreting these results, which are unlikely to reflect the true dynamic range of the biosensor when these metabolites are instead produced in the BCAA biosynthetic pathway. Measuring the biosensor response using strains engineered to produce different levels of isobutanol or isopentanol (and thus produce different levels of α-IPM and α-KIV) is a more direct approach to studying the operational range of the biosensor as it is used in real applications (Fig. 1b,d); however, this method is still limited by the maximal productivity achieved in our best engineered strains. Therefore, these experiments provide at best a conservative estimate of the true dynamic range of the biosensor.

**Supplementary Note 3: Mechanistic insights from intracellular α-IPM concentrations.**

The relationship between biosensor readout, BCHA production, and intracellular α-IPM concentration is consistent with previous reports of Leu3p activation by this metabolite[1,2], and

reveals mechanistic differences between the isobutanol and isopentanol configurations of the biosensor. We observed a significant difference in α-IPM concentrations between an isobutanol-producing strain and a negative control, starting from the exponential phase (12h), which further increases through to the stationary phase (30h), (Supplementary Figure 4a,b). However, even though the α-IPM difference is larger in the stationary phase, the difference in biosensor output is larger during the exponential phase, which is consistent with our initial observation that data from the exponential phase is more reliable and reproducible. The rapid drop in biosensor output during the stationary phase is likely due to increased GFP degradation rate, exacerbated by the PEST tag, although other factors that may contribute by repressing Leu3p or $P_{LEU1}$ activity in the stationary phase cannot be ruled out. These observations contrast with isopentanol-producing strains and the isopentanol-configured biosensor, in which the difference in α-IPM concentration between an isopentanol-producing strain and a negative control is not significant until the stationary phase measurement, yet the difference in biosensor output during the stationary phase is substantially larger than what is observed in the isobutanol-producing strain with the isobutanol-configured biosensor (Supplementary Figure 4d,e). This is consistent with the fact that *LEU2* is deleted in the isobutanol-producing strains but not in the isopentanol-producing strains, which would be expected to cause α-IPM accumulation in the former but not the later strains. It is also consistent with the fact that the GFP reporter in the isopentanol-configured biosensor does not have a PEST tag, which makes its response more sensitive to low α-IPM concentrations and longer-lasting into the stationary phase. Interestingly, the isopentanol-configured biosensor is more sensitive to small differences in α-IPM concentrations in the lag and exponential phases of fermentation than our method using U-HPLC-orbitrap MS to measure intracellular α-IPM concentrations from cell cultures. The large difference in intracellular concentrations of α-IPM between the isobutanol and

isopentanol strains, due to the different *LEU2* backgrounds, explains why the isobutanol-configured biosensor functions optimally with a PEST tag fused to the GFP reporter (to avoid high background and possibly early GFP saturation due to the higher α-IPM concentration), while the isopentanol-configured biosensor demonstrates increased sensitivity to lower α-IPM concentrations without a PEST tag.

These results also explain why biosensor measurements taken during the exponential phase are most predictive of BCHA production even though those differences are better observed in measurements taken during the stationary phase (Supplementary Figure 4a,b,d,e). During the exponential phase, both biosensor configurations display the highest sensitivity to small differences in α-IPM concentrations (as discussed above and shown in Supplementary Figure 4a,b,d,e). Conversely, while BCHA production increases dramatically by the time the fermentation reaches stationary phase, both biosensor configurations are less sensitive to even large variations in α-IPM concentrations between strains at this late stage of the fermentation (possibly due, at least in part, to increased GFP degradation and extraneous Leu3p or $P_{LEU1}$ regulation). BCHA concentrations at early to mid-exponential phase of fermentation (when biosensor readout is most predictive), are nonetheless lower (and thus more difficult to measure) than in stationary phase, likely due, at least in part, to lower cell concentrations and shorter time given to convert glucose to products in these early phases. This is especially true when comparing low cell density fermentations in 2% glucose (used in FACS to isolate high-producing strains and in the experiments shown in new Supplementary Figure 4) to high cell density fermentations in higher glucose concentrations (used in BCHAs production fermentations in most of the study). Nevertheless, biosensor outputs measured during the exponential phase can clearly predict

differences in the accumulated BCHA production after 24-48 hours of fermentation, including in high cell density fermentations. Therefore, we used biosensor measurements in the exponential phase of low cell density fermentations to isolate higher-producing strains, whose enhanced productivity we then confirmed in high cell density 48h fermentations, notably obtaining very low rates of false-positives (Supplementary Figure 15).

**Supplementary Note 4: Additional structural analysis of *ILV6* mutants that enhance isobutanol production isolated with the biosensor.** Val110, which when mutated to glutamate results in the most active variant, is located inside the putative valine binding site, interacting with the L91 residue mutated in $ILV6^{V90D/L91F}$ (Supplementary Fig. 8b). Several other mutations that increase Ilv2p activity, found multiple times in several isolated *ILV6* variants, are also located inside the valine binding pocket (N86, V90, L91, and N104), consistent with other mutations previously reported to make Ilv2p or its homologues insensitive to valine inhibition[9-13].

**Supplementary Note 5: Additional structural analysis of *LEU4* mutants that enhance isopentanol production isolated with the biosensor.** Mutating residue H541 has been previously reported to make Leu4p resistant to $Zn^{2+}$-mediated inactivation by CoA[3]. The residues corresponding to mutations Y538N, V584E, and T590I are located inside the leucine binding site, and Y485N in its vicinity suggesting that they result in decreased sensitivity to leucine inhibition. Seven residues (K51, Q439, F497, N515, V584, D578, and T590) are substituted in more than one variant and at least one of them is mutagenized in 14 of the 19 variants containing two or more mutations (Supplementary Tables 5 and 6). Residues Q439, F497, N515, V584, D578, and T590 are located in the regulatory domain (Supplementary Fig. 11), making it likely that they are also

involved in reducing regulatory inhibition of Leu4p. Most of them are located inside or in the vicinity of the regulatory leucine binding site. Remarkably, N515, located inside the leucine binding site, is substituted in five of the 24 variants we identified, including *LEU4* mutant #6, which is the variant that produces most isopentanol. On the other hand, Q439 is far from the leucine binding site but close to H541, suggesting it might be involved in $Zn^{2+}$-mediated CoA inactivation of Leu4p (Supplementary Fig. 11).

**Supplementary Note 6**: **Additional structural analysis of *Ll-ilvD* mutants that enhance cytosolic isobutanol production isolated with the biosensor.** The lobes that define the putative substrate entrance to the active site are known to undergo a significant conformational change[14]. In one conformation, the lobes seal the active site away from the solvent surrounding the enzyme (Supplementary Fig. 14c), presumably to protect the 2Fe-2S cluster in the active site from oxidation. In the other conformation, the lobes move apart from each other to open the entrance to the active site, probably to allow substrates and products to enter and exit (Supplementary Fig. 14d). This mechanism suggests that there is a balance between protecting the catalytic 2Fe-2S cluster from oxidation and allowing substrate and product exchange in the active site. Therefore, it is an intriguing possibility that mutations I433V and S189P improve enzyme activity by shifting this balance to one more optimal for the conditions in the yeast cytosol. Finally, mutation V12A, is located near the N-terminus of the enzyme, in a region involved in packing the tetramer and stabilizing the C-terminal H579, which coordinates the catalytic $Mg^{2+}$ in the active site (Supplementary Fig. 14e). It is possible that this mutation increases the stability of these structural features to enhance enzymatic activity.

**Supplementary Note 7: Supplementary methods for optogenetic experiments**. Blue LED panels (HQRP New Square 12” Grow Light Blue LED 14W, Amazon) were placed above the 24-well microtiter plates to stimulate cell growth with blue light at an intensity range of 70-90 μmol $m^{-2}$ $s^{-1}$ and duty cycles of 15 s on and 65 s off. The vertical distance between the LED panel and the top of the 24-well plates was adjusted based on the light intensity of each LED panel, measured with a spectrum quantum meter (Cat. MQ-510, Apogee Instruments, Inc., UT, USA). The light duty cycles were achieved by regulating the LED panels using a Nearpow Multifunctional Infinite Loop Programmable Plug-in Digital Timer Switch (purchased from Amazon). Single colonies were grown overnight in 1 mL of SC or SC-ura medium supplemented with 2% glucose in individual wells of the 24-well microtiter plates under constant blue light in an orbital shaker (Eppendorf, New Brunswick, USA) at 30°C and at 200 rpm agitation. The next day, each overnight culture was used to inoculate 1 mL of the same medium to reach an initial $OD_{600}$ of 0.1 and grown at 30°C, 200 rpm, and under pulsed blue light (15 s on and 65 s off). We incubated the cultures in the light until they reached different cell densities (ρ), at which we switched them from light to dark. The 24-well plates were kept in the dark by wrapping them with aluminum-foil (and continuing to incubate at 30 °C, 200 rpm) for θ hours (the incubation time in the dark). After the dark incubation period, the cells were centrifuged and re-suspended in 1 mL of fresh SC-ura medium supplemented with 2% glucose. The plates were covered with sterile adhesive SealPlate® films (Cat. # STR-SEAL-PLT; Excel Scientific, Victorville, CA) and incubated in the dark (wrapped in aluminum foil) for 48 h at 30°C and 200 rpm shaking. Subsequently, the cultures were centrifuged for 5 min at 1000 rpm, and the supernatants were collected and used for HPLC analysis.

## Supplementary Tables

**Supplementary Table 1. Yeast strains used in this study.**

| Strain | Description | Genotype (Plasmid contents in parenthesis) | Source |
|---|---|---|---|
| CEN.PK2-1C | Wild-type *Saccharomyces cerevisiae* | *MAT**a** ura3-52 trp1-289 leu2-3,112 his3-1* $MAL2\text{-}8^{c}$ *SUC2* | [15] |
| YZy81 | *bat1Δ*, isobutanol-configured biosensor (cassette from pYZ16) | CEN.PK2-1C, *his3*::*HIS3*-$P_{LEU1}$-yEGFP-PEST-$T_{ADH1}$-$P_{TPI1}$-$LEU4^{1\text{-}410}$-$T_{PGK1}$, *bat1Δ*::hphMX | This study |
| YZy90 | *pdc1Δ, pdc5Δ, pdc6Δ*, *gal80Δ*, pJLA121-$PDC1^{0202}$ | CEN. PK2-1C, *pdc1Δ, pdc5Δ, pdc6Δ, gal80Δ*::lox71-kanMX-lox66, 2μ *URA3* plasmid ($P_{TFE1}$-*PDC1*-$T_{ACT1}$) | This study |
| YZy91 | *bat1Δ, bat2Δ, ilv6Δ*, isobutanol-configured biosensor (cassette from pYZ16) | CEN.PK2-1C, *his3*::*HIS3*-$P_{LEU1}$-yEGFP-PEST-$T_{ADH1}$-$P_{TPI1}$-$LEU4^{1\text{-}410}$-$T_{PGK1}$, *bat1Δ*::hphMX *bat2Δ*::lox71-kanMX-lox66 *ilv6Δ*::lox71-natMX-lox66 | This study |
| YZy121 | CEN.PK2-1C, isobutanol-configured biosensor (cassette from pYZ16) | CEN.PK2-1C, *his3*::*HIS3*-$P_{LEU1}$-yEGFP-PEST-$T_{ADH1}$-$P_{TPI1}$-$LEU4^{1\text{-}410}$-$T_{PGK1}$ | This study |
| YZy140 | *bat1Δ, leu4Δ, leu9Δ, LEU2* restored | CEN.PK2-1C, *bat1Δ*::hphMX *leu4Δ*::lox71-kanMX-lox66 *leu9Δ*::lox71-natMX-lox66 *leu2::LEU2* | This study |
| YZy148 | *bat1Δ, leu4Δ, leu9Δ, LEU2* restored, modified isopentanol-configured biosensor (cassette from pYZ24) | CEN. PK2-1C, *his3*::*HIS3*-$P_{LEU1}$-yEGFP-$T_{ADH1}$ *bat1Δ*::hphMX *leu4Δ*::lox71-kanMX-lox66 *leu9Δ*::lox71-natMX-lox66 *leu2*::*LEU2* | This study |
| YZy230 | YZy121, cassette from pYZ33 (Strain 1) | YZy121, δ-integration-$P_{TDH3}$-*ILV2*_cHATag-$T_{ADH1}$, $P_{PGK1}$-*ILV3*_cHisTag-$T_{CYC1}$, $P_{TEF1}$-*ILV5*_cMycTag-$T_{ACT1}$ | This study |
| YZy231 | YZy121, cassette from pYZ33 (Strain 2) | YZy121, δ-integration-$P_{TDH3}$-*ILV2*_cHATag-$T_{ADH1}$, $P_{PGK1}$-*ILV3*_cHisTag-$T_{CYC1}$, $P_{TEF1}$-*ILV5*_cMycTag-$T_{ACT1}$ | This study |

| Strain | Description | Genotype (Plasmid contents in parenthesis) | Source |
|---|---|---|---|
| YZy232 | YZy121, cassette from pYZ33 (Strain 3) | YZy121, δ-integration-$P_{TDH3}$-*ILV2*-cHATag-$T_{ADH1}$-$P_{PGK1}$-*ILV3*-cHisTag-$T_{CYC1}$-$P_{TEF1}$-*ILV5*-cMycTag-$T_{ACT1}$ | This study |
| YZy233 | YZy81, cassette from pYZ33 (Strain 1) | YZy81, δ-integration-$P_{TDH3}$-*ILV2*-cHATag-$T_{ADH1}$-$P_{PGK1}$-*ILV3*-cHisTag-$T_{CYC1}$-$P_{TEF1}$-*ILV5*-cMycTag-$T_{ACT1}$ | This study |
| YZy234 | YZy81, cassette from pYZ33 (Strain 2) | YZy81, δ-integration-$P_{TDH3}$-*ILV2*-cHATag-$T_{ADH1}$-$P_{PGK1}$-*ILV3*-cHisTag-$T_{CYC1}$-$P_{TEF1}$-*ILV5*-cMycTag-$T_{ACT1}$ | This study |
| YZy235 | YZy81, cassette from pYZ34 (Strain 1) | YZy81, δ-integration-$P_{TDH3}$-*ILV2*-cHATag-$T_{ADH1}$-$P_{PGK1}$-*ILV3*-cHisTag-$T_{CYC1}$-$P_{TEF1}$-CoxIV$_{MLS}$-*Ll_adhA*$^{RE1}$-cMycTag-$T_{ACT1}$-[$P_{TDH3}$-CoxIV$_{MLS}$-*ARO10*-cHATag-$T_{ADH1}$-$P_{TEF1}$-*ILV5*-cMycTag-$T_{ACT1}$] | This study |
| YZy236 | YZy81, cassette from pYZ34 (Strain 2) | YZy81, δ-integration-$P_{TDH3}$-*ILV2*-cHATag-$T_{ADH1}$-$P_{PGK1}$-*ILV3*-cHisTag-$T_{CYC1}$-$P_{TEF1}$-CoxIV$_{MLS}$-*Ll_adhA*$^{RE1}$-cMycTag-$T_{ACT1}$-[$P_{TDH3}$-CoxIV$_{MLS}$-*ARO10*-cHATag-$T_{ADH1}$-$P_{TEF1}$-*ILV5*-cMycTag-$T_{ACT1}$] | This study |
| YZy311 | YZy91, pYZ127 | YZy91, CEN *URA3* plasmid ($P_{TDH3}$-*ILV6*-$T_{ADH1}$) | This study |
| YZy312 | YZy91, pYZ228 | YZy91, CEN *URA3* plasmid ($P_{TDH3}$-*ILV6*$^{V110E}$-$T_{ADH1}$) | This study |
| YZy313 | YZy363, pYZ127 | YZy363, CEN *URA3* plasmid ($P_{TDH3}$-*ILV6*-$T_{ADH1}$) | This study |
| YZy314 | YZy363, pYZ228 | YZy363, CEN *URA3* plasmid ($P_{TDH3}$-*ILV6*$^{V110E}$-$T_{ADH1}$) | This study |
| YZy363 | YZy81, overexpressing the mitochondrial isobutanol pathway via δ-integration using cassette from pYZ34 | YZy81, δ-integration-$P_{TDH3}$-*ILV2*-cHATag-$T_{ADH1}$-$P_{PGK1}$-*ILV3*-cHisTag-$T_{CYC1}$-$P_{TEF1}$-CoxIV$_{MLS}$-*Ll_adhA*$^{RE1}$-cMycTag-$T_{ACT1}$-[$P_{TDH3}$-CoxIV$_{MLS}$-*ARO10*-cHATag-$T_{ADH1}$-$P_{TEF1}$-*ILV5*-cMycTag-$T_{ACT1}$] | This study |
| YZy418 | *ilv3Δ,* isobutanol-configured biosensor (cassette from pYZ16) | CEN.PK2-1C, *his3*::*HIS3*-$P_{LEU1}$-yEGFP-PEST-$T_{ADH1}$-$P_{TPI1}$-*LEU4*$^{1\text{-}410}$-$T_{PGK1}$, *ilv3Δ*:: lox71-hphMX-lox66 | This study |
| YZy443 | YZy418, *tma29Δ* | YZy418, *tma29Δ*::lox71-kanMX-lox66 | This study |

| Strain | Description | Genotype (Plasmid contents in parenthesis) | Source |
|---|---|---|---|
| YZy447 | YZy443, cytosolic isobutanol pathway (cassette from pYZ196) | YZy443, *ura3Δ*::loxP-*URA3*-loxP-$P_{TDH3}$-*AFT1*-$T_{ADH1}$-[$P_{TEF1}$-*Bs_alsS*-$T_{ACT1}$-$P_{TDH3}$-*Ec_ilvC*$^{P2D1-A1}$-$T_{ADH1}$]-$P_{GAL10}$-*Ll_ilvD*-$T_{ACT1}$ | This study |
| YZy449 | YZy447, *ura3Δ* marker restored | YZy447, *ura3*Δ | This study |
| YZy452 | YZy449, (cassette from pYZ206) | YZy449, δ-integration-$P_{TDH3}$-*Ec_ilvC*$^{P2D1-A1}$-$T_{ADH1}$-[$P_{TEF1}$- *Ec_ilvC*$^{P2D1-A1}$-$T_{ACT1}$] | This study |
| YZy453 | YZy452, pYZ125 | YZy452, empty CEN *URA3* plasmid | This study |
| YZy454 | YZy452, pYZ126 | YZy452, CEN *URA3* plasmid ($P_{TDH3}$-*Ll_ilvD*-$T_{ADH1}$) | This study |
| YZy468 | YZy452, pYZ341 | YZy452, 2μ *URA3* plasmid ($P_{TDH3}$-*Ll_ilvD*-$T_{ADH1}$) | This study |
| YZy469 | YZy452, pYZ353 | YZy452, CEN *URA3* plasmid ($P_{TDH3}$-*Ll_ilvD*$^{I433V}$-$T_{ADH1}$) | This study |
| YZy470 | YZy452, pYZ342 | YZy452, 2μ *URA3* plasmid ($P_{TDH3}$-*Ll_ilvD*$^{I433V}$-$T_{ADH1}$) | This study |
| YZy480 | YZy90, OptoINVRT7, 2μ plasmid pJLA121- *PDC1*$^{0202}$ | YZy90, *his3*::*HIS3*-$P_{TEF1}$-EL222-$T_{CYC1}$-$P_{C120}$-*GAL80*-ODC$^{mut}$-$T_{ACT1}$-[$P_{C120}$-*GAL80*-ODC$^{mut}$-$T_{ACT1}$]-$P_{PGK1}$-*GAL4*-PSD$^{V19L}$-$T_{ADH1}$, 2μ *URA3* plasmid ($P_{TEF1}$-*PDC1*-$T_{ACT1}$) | This study |
| YZy481 | YZy90, OptoINVRT7, 2μ plasmid pJLA121- *PDC1*$^{0202}$ removed | YZy90, *his3*::*HIS3*-$P_{TEF1}$-EL222-$T_{CYC1}$-$P_{C120}$-*GAL80*-ODC$^{mut}$-$T_{ACT1}$-[$P_{C120}$-*GAL80*-ODC$^{mut}$-$T_{ACT1}$]-$P_{PGK1}$-*GAL4*-PSD$^{V19L}$-$T_{ADH1}$ | This study |
| YZy487 | YZy481, isobutanol-configured biosensor integrated to *GAL80* locus (using the cassette from pYZ414) | YZy481, *gal80*::Lox71-natMX6-Lox66-$P_{LEU1}$-yEGFP-PEST-$T_{ADH1}$-$P_{TPI1}$-*LEU4*$^{1-410}$-$T_{PGK1}$ | This study |
| YZy502 | YZy487, OptoEXP *PDC1* and OptoINVRT7 cytosolic isobutanol pathway | YZy487, δ-integration-$P_{C120}$-*PDC1*-$T_{ACT1}$-$P_{TDH3}$-*Ec_ilvC*$^{P2D1-A1}$-$T_{CYC1}$-$P_{TEF1}$-*Ll_ilvD*$^{I433V}$-$T_{TPS1}$-$P_{GAL1-S}$-*Bs_alsS*-$T_{ACT1}$ | This study |

| Strain | Description | Genotype (Plasmid contents in parenthesis) | Source |
|---|---|---|---|
| YZy505 | YZy502, partial cytosolic isobutanol pathway lacking *Bs_alsS* (pYZ350) | YZy502, 2µ *URA3* plasmid ($P_{TDH3}$-*Ec_ilvC*$^{P2D1\text{-}A1}$-$T_{CYC1}$-[$P_{TEF1}$-*Ec_ilvC*$^{P2D1\text{-}A1}$-$T_{ACT1}$-$P_{TDH3}$-*Ll_ilvD*$^{I433V}$-$T_{ADH1}$]-$P_{PGK1}$-*ARO10*-cHATag-$T_{CYC1}$-$P_{TEF1}$-*Ll_adhA*$^{RE1}$-cMycTag-$T_{ACT1}$) | This study |
| SHy1 | *bat1Δ* | CEN.PK2-1C, *bat1Δ*::hphMX | [13] |
| SHy134 | *bat1Δ, leu4Δ, leu9Δ, LEU2* restored, isopentanol-configured biosensor (cassette from pYZ25) | CEN.PK2-1C, *his3*::*HIS3*-$P_{LEU1}$-yEGFP-$T_{ADH1}$-$P_{TPI1}$-*LEU4*$^{\Delta S547}$-$T_{PGK1}$ *bat1Δ*::hphMX *leu4Δ*::lox71-kanMX-lox66 *leu9Δ*::lox71-natMX-lox66 *leu2::LEU2* | This study |
| SHy158 | SHy134, pYZ125 | SHy134, empty CEN *URA3* plasmid | This study |
| SHy159 | SHy134, JLAb691 | SHy134, CEN *URA3* plasmid ($P_{TDH3}$-*ILV2*_cHATag-$T_{ADH1}$, $P_{PGK1}$-*ILV3*_cHisTag-$T_{CYC1}$, $P_{TEF1}$-*ILV5*_cMycTag-$T_{ACT1}$) | This study |
| SHy176 | SHy134, JLAb705 | SHy134, CEN *URA3* plasmid ($P_{TDH3}$-*ILV1*_cHATag-$T_{ADH1}$) | This study |
| SHy181 | *leu4Δ, leu9Δ, LEU2* restored, *BAT1* restored, isopentanol-configured biosensor (cassette from pYZ25) | CEN. PK2-1C, *his3*::*HIS3*-$P_{LEU1}$-yEGFP-$T_{ADH1}$-$P_{TPI1}$-*LEU4*$^{\Delta S547}$-$T_{PGK1}$ *leu4Δ*::lox71-kanMX-lox66 *leu9Δ*::lox71-natMX-lox66 *leu2*::*LEU2 trp1*::*TRP1*-$P_{BAT1}$-*BAT1*-$T_{BAT1}$ | This study |
| SHy187 | SHy181, pYZ125 | SHy181, empty CEN *URA3* plasmid | This study |
| SHy188 | SHy181, JLAb691 | SHy181, CEN *URA3* plasmid ($P_{TDH3}$-*ILV2*_cHATag-$T_{ADH1}$, $P_{PGK1}$-*ILV3*_cHisTag-$T_{CYC1}$, $P_{TEF1}$-*ILV5*_cMycTag-$T_{ACT1}$) | This study |
| SHy192 | SHy181, JLAb705 | SHy181, CEN *URA3* plasmid ($P_{TDH3}$-*ILV1*_cHATag-$T_{ADH1}$) | This study |

**Supplementary Table 2. Plasmids used in this study.**

| Plasmid | Description [Brackets indicate inverted orientation] | Source |
|---|---|---|
| pRS416 | $Amp^R$, CEN, URA3 | [16] |
| pRS426 | $Amp^R$, 2μ, URA3 | [17] |
| pYZ1 | $Amp^R$, 2μ, TRP1, $P_{TPI1}$-$LEU4^{\Delta S547}$_FLAG-$T_{PGK1}$ | This study |
| pYZ2 | $Amp^R$, 2μ, TRP1, $P_{TPI1}$-$LEU4^{1\text{-}410}$_FLAG-$T_{PGK1}$ | This study |
| pYZ12B | $Amp^R$, *HIS3* locus integration vector (His3INT) | [18] |
| pYZ13 | $Amp^R$, 2μ, URA3, $P_{LEU1}$-yEGFP_PEST-$T_{ADH1}$ | This study |
| pYZ14 | $Amp^R$, His3INT, $P_{LEU1}$-yEGFP_PEST-$T_{ADH1}$ | This study |
| pYZ15 | $Amp^R$, His3INT, $P_{LEU1}$-yEGFP_PEST-$T_{ADH1}$, $P_{TPI1}$-$LEU4^{\Delta S547}$_FLAG-$T_{PGK1}$ | This study |
| pYZ16 | (Isobutanol-configured biosensor)<br>$Amp^R$, His3INT, $P_{LEU1}$-yEGFP_PEST-$T_{ADH1}$-$P_{TPI1}$-$LEU4^{1\text{-}410}$_FLAG-$T_{PGK1}$ | This study |
| pYZ17 | $Amp^R$, Lox71-kanMX-Lox66 gene-disruption cassette | [19] |
| pYZ23 | $Amp^R$, δ-integration vector, Lox71-bleMX6-Lox66 | [18] |
| pYZ24 | (Modified isobutanol-configured biosensor without leucine-insensitive Leu4p mutant)<br>$Amp^R$, His3INT, $P_{LEU1}$-yEGFP-$T_{ADH1}$ | This study |
| pYZ25 | (Isopentanol-configured biosensor)<br>$Amp^R$, His3INT, $P_{LEU1}$-yEGFP-$T_{ADH1}$-$P_{TPI1}$-$LEU4^{\Delta S547}$_FLAG-$T_{PGK1}$ | This study |
| pYZ26 | $Amp^R$, His3INT, $P_{LEU1}$-yEGFP-$T_{ADH1}$-$P_{TPI1}$-$LEU4^{1\text{-}410}$_FLAG-$T_{PGK1}$ | This study |

| | | |
|---|---|---|
| pYZ33 | (δ-integration-*ILV*s)<br>Amp$^{R}$, δ-integration-Lox71-ShBle-Lox66-P$_{TDH3}$-*ILV2*-cHATag-T$_{ADH1}$-P$_{PGK1}$-*ILV3*-cHisTag-T$_{CYC1}$-P$_{TEF1}$-*ILV5*-cMycTag-T$_{ACT1}$ | [4] |
| pYZ34 | (δ-integration-ILVs, CoxIV$_{MLS}$-*ARO10*, CoxIV$_{MLS}$-*Ll_adhA*$^{RE1}$)<br>Amp$^{R}$, δ-integration-Lox71-ShBle-Lox66-P$_{TDH3}$-*ILV2*-cHATag-T$_{ADH1}$-P$_{PGK1}$-*ILV3*-cHisTag-T$_{CYC1}$-P$_{TEF1}$-CoxIV$_{MLS}$-*Ll_adhA*$^{RE1}$-cMycTag-T$_{ACT1}$-[P$_{TDH3}$-CoxIV$_{MLS}$-*ARO10*-cHATag-T$_{ADH1}$-P$_{TEF1}$-*ILV5*-cMycTag-T$_{ACT1}$] | [19] |
| pYZ55 | Amp$^{R}$, Lox71-hphMX-Lox66 gene-disruption cassette | [19] |
| pYZ57 | Amp$^{R}$, His3INT, P$_{LEU1}$-yEGFP-T$_{ADH1}$-P$_{TPI1}$-*LEU4*$^{1\text{-}474}$_FLAG-T$_{PGK1}$ | This study |
| pYZ61 | Amp$^{R}$, His3INT, P$_{LEU1}$-yEGFP_PEST-T$_{ADH1}$, P$_{TPI1}$-*LEU4*$^{1\text{-}474}$_FLAG-T$_{PGK1}$ | This study |
| pYZ84 | Amp$^{R}$, Lox71-natMX-Lox66 gene-disruption cassette | [19] |
| pYZ113 | (δ-integration-CoxIV$_{MLS}$-*ARO10*, CoxIV$_{MLS}$-*Ll_adhA*$^{RE1}$)<br>Amp$^{R}$, δ-integration-Lox71-ShBle-Lox66-P$_{TDH3}$-CoxIV$_{MLS}$-*ARO10*-cHATag-T$_{ADH1}$-[P$_{TEF1}$-CoxIV$_{MLS}$-*Ll_adhA*$^{RE1}$-cMycTag-T$_{ACT1}$] | This study |
| pYZ125 | Amp$^{R}$, CEN, URA3, P$_{TDH3}$-MCS-T$_{ADH1}$ | This study |
| pYZ126 | Amp$^{R}$, CEN, URA3, P$_{TDH3}$-*Ll_ilvD*-T$_{ADH1}$ | This study |
| pYZ127 | Amp$^{R}$, CEN, URA3, P$_{TDH3}$-*ILV6*-T$_{ADH1}$ | This study |
| pYZ148 | Amp$^{R}$, CEN, URA3, P$_{TDH3}$-*ILV6*$^{V90D/L91F}$-T$_{ADH1}$ | This study |
| pYZ149 | Amp$^{R}$, CEN, URA3, P$_{TDH3}$-*LEU4*-T$_{ADH1}$ | This study |
| pYZ154 | Amp$^{R}$, CEN, URA3, P$_{TDH3}$-*LEU4*$^{\Delta S547}$-T$_{ADH1}$ | This study |
| pYZ155 | Amp$^{R}$, CEN, URA3, P$_{TDH3}$-*LEU4*$^{1\text{-}410}$-T$_{ADH1}$ | This study |
| pYZ196 | (Cytosolic isobutanol pathway containing P$_{GAL10}$-*Ll_ilvD*) | This study |

| | | |
|---|---|---|
| | $Amp^R$, Ura3 Locus integration-LoxP-*URA3*-LoxP- $P_{TDH3}$-*AFT1*-$T_{ADH1}$-[$P_{TEF1}$-*Bs_alsS*-$T_{ACT1}$-$P_{TDH3}$-*Ec_ilvC*$^{P2D1-A1}$-$T_{ADH1}$]-$P_{GAL10}$-*Ll_ilvD*-$T_{ACT1}$ | |
| pYZ206 | (δ-integration-two copies of *Ec_ilvC*$^{P2D1-A1}$)<br>$Amp^R$, δ-integration-Lox71-ShBle-Lox66-$P_{TDH3}$-*Ec_ilvC*$^{P2D1-A1}$-$T_{ADH1}$-[$P_{TEF1}$-*Ec_ilvC*$^{P2D1-A1}$-$T_{ACT1}$] | This study |
| pYZ223 | $Amp^R$, Lox71-natMX6-Lox66, *GAL80* Locus Integration vector (Gal80INT-Lox71-natMX6-Lox66) | This study |
| pYZ228 | $Amp^R$, CEN, URA3, $P_{TDH3}$-*ILV6*$^{V110E}$-$T_{ADH1}$ | This study |
| pYZ341 | $Amp^R$, 2μ, URA3, $P_{TDH3}$-*Ll_ilvD*-$T_{ADH1}$ | This study |
| pYZ342 | $Amp^R$, 2μ, URA3, $P_{TDH3}$-*Ll_ilvD*$^{I433V}$-$T_{ADH1}$ | This study |
| pYZ350 | (Partial cytosolic isobutanol pathway containing *ARO10, Ll_adhA*$^{RE1}$, *Ll_ilvD*$^{I433V}$, and extra copies of *Ec_ilvC*$^{P2D1-A1}$; lacking *Bs_alsS*)<br>$Amp^R$, 2μ, URA3, $P_{TDH3}$-*Ec_ilvC*$^{P2D1-A1}$-$T_{CYC1}$-[$P_{TEF1}$-*Ec_ilvC*$^{P2D1-A1}$-$T_{ACT1}$]-[$P_{TDH3}$-*Ll_ilvD*$^{I433V}$-$T_{ADH1}$]-$P_{PGK1}$-*ARO10*-cHATag-$T_{CYC1}$-$P_{TEF1}$-*Ll_adhA*$^{RE1}$-cMycTag-$T_{ACT1}$ | This study |
| pYZ353 | $Amp^R$, CEN, URA3, $P_{TDH3}$-*Ll_ilvD* $^{I433V}$-$T_{ADH1}$ | This study |
| pYZ383 | $Amp^R$, 2μ, URA3, $P_{TEF1}$-*Ll_ilvD*$^{I433V}$-$T_{TPS1}$ | This study |
| pYZ384 | $Amp^R$, 2μ, URA3, $P_{Gal1-S}$-*Bs_alsS*-$T_{ACT1}$ | This study |
| pYZ414 | $Amp^R$, Gal80INT-Lox71-natMX6-Lox66-Isobutanol-configured biosensor ($P_{LEU1}$-yEGFP_PEST-$T_{ADH1}$-$P_{TPI1}$-*LEU4*$^{1-410}$_FLAG-$T_{PGK1}$) | This study |
| pYZ417 | (δ-integration-OptoEXP-*PDC1*-OptoINVRT7-cytosolic isobutanol pathway)<br>$Amp^R$, δ-integration-Lox71-ShBle-Lox66-OptoEXP-*PDC1* ($P_{C120}$-*PDC1*-$T_{ACT1}$)-OptoINVRT7-cytosolic isobutanol pathway ($P_{TDH3}$- *Ec_ilvC*$^{P2D1-A1}$-$T_{CYC1}$-$P_{TEF1}$-*Ll_ilvD*$^{I433V}$-$T_{TPS1}$-$P_{Gal1-S}$-*Bs_alsS*-$T_{ACT1}$) | This study |
| EZ-L235 | (δ-integration-OptoEXP-*PDC1*)<br>$Amp^R$, δ-integration-Lox71-ShBle-Lox66-OptoEXP-*PDC1* ($P_{C120}$-*PDC1*-$T_{ACT1}$) | [18] |

| | | |
|---|---|---|
| EZ-L439 | (OptoINVRT7)<br>Amp$^R$, His3INT-P$_{TEF1}$-EL222-T$_{CYC1}$-P$_{C120}$-*GAL80*-ODC$^{mut}$-T$_{ACT1}$-[P$_{C120}$-*GAL80*-ODC$^{mut}$-T$_{ACT1}$]-P$_{PGK1}$-*GAL4*-PSD$^{V19L}$ -T$_{ADH1}$ | [20] |
| pAG26 | Amp$^R$, Plasmid containing hphMX gene-disruption cassette | [21] |
| pJA123 | Amp$^R$, 2μ, URA3, P$_{TDH3}$-*ILV2*-cHATag-T$_{ADH1}$-P$_{PGK1}$-*ILV3*-cHisTag-T$_{CYC1}$-P$_{TEF1}$-*ILV5*-cMycTag-T$_{ACT1}$ | [22] |
| pJA182 | Amp$^R$, 2μ, URA3, P$_{TDH3}$-*ILV2*-HA-T$_{ADH1}$-P$_{PGK1}$-*ILV3*-cHisTag-T$_{CYC1}$-P$_{TEF1}$-CoxIV$_{MLS}$-*Ll_adhA$^{RE1}$*-cMycTag-T$_{ACT1}$-[P$_{TDH3}$-CoxIV$_{MLS}$-*ARO10*-cHATag-T$_{ADH1}$-P$_{TEF1}$-*ILV5*-cMycTag-T$_{ACT1}$] | [22] |
| pJA248 | Amp$^R$, Plasmid containing yEGFP and PEST protein degradation tag | This study |
| pJLA121-*PDC1*$^{0202}$ | Amp$^R$, 2μ, URA3, P$_{TFE1}$-*PDC1*-T$_{ACT1}$ | [18] |
| JLAb23 | Amp$^R$, 2μ, URA3, pRS426-P$_{TPI1}$-MCS_FLAG-T$_{PGK1}$ | This study |
| JLAb131 | Amp$^R$, 2μ, URA3, pJLA121$^{0103}$- P$_{TDH3}$-MCS-T$_{ADH1}$ | This study |
| JLAb581 | Amp$^R$, 2μ, URA3, P$_{TDH3}$-CoxIV$_{MLS}$-*ARO10*-cHATag-T$_{ADH1}$-[P$_{TEF1}$- CoxIV$_{MLS}$-*Ll_adhA$^{RE1}$*-cMycTag-T$_{ACT1}$] | [13] |
| JLAb691 | Amp$^R$, CEN, URA3, P$_{TDH3}$-*ILV2*_cHATag-T$_{ADH1}$-P$_{PGK1}$-*ILV3*_cHisTag-T$_{CYC1}$-P$_{TEF1}$-*ILV5*_cMycTag-T$_{ACT1}$ | This study |
| JLAb705 | Amp$^R$, CEN, URA3, P$_{TDH3}$-*ILV1*_cHATag-T$_{ADH1}$ | This study |
| pSH63 | Amp$^R$, CEN, TRP1, P$_{GAL1}$_Cre_T$_{CYC1}$ | [23] |

**Supplementary Table 3**. Digital droplet PCR to identify the average cassette integration genotype of the pre-sorted library (PSL) and genotypes of the six colonies producing ~700 mg/L of isobutanol or above (Y436-Y443), isolated after two rounds of FACS. Source data are provided as a Source Data file.

| | Positive counts | | | | Cassette Integrations | | |
|---|---|---|---|---|---|---|---|
| **GDNA** | **A + C** | **B + C** | **A + B + C** | **R** | **A** | **B** | **C** |
| **CEN.PK2** | 0 | 362 | 0 | 448 | n.a | n.a | n.a |
| **PSL** | 436 | 440 | 544 | 494 | 0.21 | 0.22 | 0.67 |
| **Y436** | 1160 | 532 | 1236 | 690 | 2 | 0 | 0 |
| **Y437** | 1150 | 598 | 1252 | 768 | 2 | 0 | 0 |
| **Y438** | 1008 | 544 | 1112 | 570 | 2 | 0 | 0 |
| **Y439** | 1026 | 484 | 1072 | 576 | 2 | 0 | 0 |
| **Y442** | 632 | 1146 | 1298 | 672 | 1 | 1 | 0 |
| **Y443** | 524 | 1064 | 1030 | 538 | 1 | 1 | 0 |

A = Upstream pathway cassette (*ILV2, ILV5, ILV3*)
B = Downstream pathway cassette (*ARO10, Ll_adhA*$^{RE1}$)
C = Complete pathway cassette (*ILV2, ILV5, ILV3, ARO10, Ll_adhA*$^{RE1}$)
R = Single copy genome reference
n.a. = Not applicable

Cassette integrations were obtained by solving the following system of equations, where X is the copy number of the zeocin resistance cassette, Y is the copy number of *ILV2*, and Z is the copy number of *ARO10* measured by ddPCR (see methods):

$$A + B + C = X \qquad \text{(Eq. 1)}$$
$$A + C = Y \qquad \text{(Eq. 2)}$$
$$B + C + 1 = Z \qquad \text{(Eq. 3)}$$

**Supplementary Table 4.** Amino acid mutations found in the sequenced Ilv6p variants.

| Mutants | Mutations |
|---|---|
| **Derived from wild-type *ILV6* random mutagenesis library** | |
| ILV6_mutant_1 | N104S |
| ILV6_mutant_2 | S71P_N86T |
| ILV6_mutant_3 | N86T |
| ILV6_mutant_4 | V90A |
| ILV6_mutant_5 | M21I_Y34C_N223Y |
| ILV6_mutant_6 | V110E |
| ILV6_mutant_7 | N86Y |
| ILV6_mutant_8 | L91S |
| ILV6_mutant_9 | N104S_E133V_S184F_G233E |
| ILV6_mutant_10 | M21I_N57S_V79D_E133G_L161Q_T185A_N186S_D224Y_K284E_E288K |
| **Derived from $ILV6^{V90D/L91F}$ random mutagenesis library** | |
| ILV6_mutant_11 | V90D_L91F_D292N |
| ILV6_mutant_12 | V90D_L91F_H219L |
| ILV6_mutant_13 | V90D_L91F_N112S |
| ILV6_mutant_14 | V90D_L91F_T185A |
| ILV6_mutant_15 | N86D_V90D_L91F |
| ILV6_mutant_16 | N86S_V90D_L91F_K202M |
| ILV6_mutant_17 | V90D_L91F_F174S |
| ILV6_mutant_18 | V90D_L91F_N153Y_E198G |
| ILV6_mutant_19 | P47T_Q85R_V90D_L91F_H180R |
| ILV6_mutant_20 | V90D_L91F_V267A |
| ILV6_mutant_21 | V90D_L91F_Q126L_S286R |
| ILV6_mutant_22 | V90D_L91F_H180Y_I239V |
| ILV6_mutant_23 | R11H_V84M_V90D_L91F_F174I |
| ILV6_mutant_24 | V90D_L91F_Q193R |

**Supplementary Table 5.** Amino acid mutations found in the sequenced Leu4p variants.

| Mutants | Mutations |
|---|---|
| **Derived from mutagenesis library of full-length *LEU4*** | |
| LEU4_mutant_1 | Y203F_V425D_N515I |
| LEU4_mutant_2 | K25N_A60T_T316N_L330M_D433G_K467R_F497L_D509G_N515D_L529M_A552V_I602L |
| LEU4_mutant_3 | N72S_K97R_S126G_R344H_R495G_N515D |
| LEU4_mutant_4 | V446A_G544C_N593Y |
| LEU4_mutant_5 | H541R |
| LEU4_mutant_6 | E86D_K191N_K374R_A445T_S481R_N515I_A568V_S601A |
| LEU4_mutant_7 | T287A_P400L_N515H_N537T |
| LEU4_mutant_8 | A144T_N240S_D578N |
| LEU4_mutant_9 | K90M_Q439R_S542P |
| LEU4_mutant_10 | R428G_N486D |
| LEU4_mutant_11 | K51R_E233K_F377I_L427S_K458E_F497I_E577D |
| LEU4_mutant_12 | R392G_S459L_V584A |
| LEU4_mutant_13 | K51R_A182V_V198T_A551V |
| **Derived from mutagenesis library of regulatory domain** | |
| LEU4_mutant_14 | Y485N |
| LEU4_mutant_15 | A450S_D451G_D578E |
| LEU4_mutant_16 | T590I_P603S |
| LEU4_mutant_17 | K489E_V573A |
| LEU4_mutant_18 | Y538N |
| LEU4_mutant_19 | Q447R_Q478H_G516D |
| LEU4_mutant_20 | V584E |
| LEU4_mutant_21 | Q439H_D581G |
| LEU4_mutant_22 | R436K_S443Y |
| LEU4_mutant_23 | D564E_T590I |
| LEU4_mutant_24 | T590I |

**Supplementary Table 6.** Occurrence and description of the most relevant mutations found in the Leu4p variants.

| Position | Number of occurrences | Variants | Single or multiple mutant variant# | Number of substitutions | Type of substitutions |
|---|---|---|---|---|---|
| **N515** | 5 | 1, 2, 3, 6, 7 | Multiple | 3 | I, D, H |
| **T590** | 3 | 16, 23, 24 | Single and Multiple | 1 | I |
| **K51** | 2 | 11, 13 | Multiple | 1 | R |
| **Q439** | 2 | 9, 21 | Multiple | 2 | R, H |
| **F497** | 2 | 2, 11 | Multiple | 2 | L, I |
| **D578** | 2 | 8, 15 | Multiple | 2 | N, E |
| **V584** | 2 | 12, 20 | Single and Multiple | 2 | A, E |
| **Y485** | 1 | 14 | Single | 1 | N |
| **Y538** | 1 | 18 | Single | 1 | N |
| **H541** | 1 | 5 | Single | 1 | R |

# Mutations observed in variants with only one mutation (Single), as one of the mutations in variants with multiple mutations (Multiple), or in both types of variants (Single and Multiple).

**Supplementary Table 7.** Published kinetic parameters of *Bs*_AlsS, *Ec*_IlvC$^{P2D1-A1}$, and *Ll*_IlvD.

| Enzymes | Specific activity (U/mg) | $K_m$ (mM) | $k_{cat}$ ($s^{-1}$) | $k_{cat}/K_m$ | Assay conditions | References |
|---|---|---|---|---|---|---|
| *Bs*_AlsS | 8.28 | 13.6 ± 0.8 | 121 ± 13 | 8.9 + 1.1 | pH 7.0, 37°C | [24] |
| *Ec*_IlvC$^{P2D1-A1}$ | n.a. | n.a. | 4.3 ± 0.3 | | pH 7.0, n.a. | [25] |
| *Ll*_IlvD | 0.62 ±0.01* | n.a. | n.a. | | n.a. | [26] |

* The highest value reported. n.a.: not available

**Supplementary Table 8.** Amino acid mutations found in the sequenced *Ll*_IlvD variants.

| **Mutants** | **Mutations** |
|---|---|
| *Ll*_ilvD_mutant_1 | I433V |
| *Ll*_ilvD_mutant_2 | V12A, S189P, H439R |
| *Ll*_ilvD_mutant_3 | K535R |
| *Ll*_ilvD_mutant_4 | K16R |
| *Ll*_ilvD_mutant_5 | E13G, K345M, I514N |
| *Ll*_ilvD_mutant_6 | I154V, I312T |

**Supplementary Table 9.** Oligonucleotides used in this study.

| Oligo Name | Sequence | Description |
|---|---|---|
| Yfz_Oli31 | GCTGGAGCTCACCGGTATACCCGGGAATATGAACCACAGTACATCATATTAAGACGTAGT | $P_{LEU1}$_Gibson_primer_F |
| Yfz_Oli32 | GAATAATTCTTCACCTTTAGACATGATTTAAAACAGCAAATAATAAAAATCGATAGCGAC | $P_{LEU1}$_Gibson_primer_R |
| Yfz_Oli33 | CGATTTTTATTATTTGCTGTTTTAAATCATGTCTAAAGGTGAAGAATTATTCACTGGTG | yEGFP_PEST-Gibson_Primer_F |
| Yfz_Oli34 | TCGCTGATCATTACTCGAGGTCGACCTATATTACTTGGGTATTGCCCATACC | yEGFP_PEST-Gibson_Primer_R |
| Yfz_Oli35 | CATGGCTAGCGTTAAAGAGAGTATTATTGC | NheI-ScLEU4$^{1\text{-}410}$ Primer-F |
| Yfz_Oli36 | ATAATCCTCGAGGACAGCTTCGTAATCACGGC | XhoI-ScLEU4$^{1\text{-}410}$ Primer-R |
| Yfz_Oli37 | CTGAGCGGCCGCTAAAATCATGGCTAGCGTTAAAGAGAGTATTATTGC | NotI-KOZAK-NheI-ScLEU4$^{WT}$ Primer-F |
| Yfz_Oli38 | ATAATCCTCGAGTGCAGAGCCAGATGCCGCAGCATTCTTA | XhoI-ScLEU4$^{WT}$ Primer-R |
| Yfz_Oli59 | TCGACACGCGTTTATTT | Annealed oligo cloning linker SalI_MluI_BsrGI_F |
| Yfz_Oli60 | GTACAAATAAACGCGTG | Annealed oligo cloning linker SalI_MluI_BsrGI_R |

| | | |
|---|---|---|
| Yfz_Oli198 | CCGCTAAAATCATGGCTAGC | Error-prone PCR universal_Primer_F for full ORF subcloned into pYZ125 |
| Yfz_Oli242 | CATAAATCATAAGAAATTCGCTGATCATT ACTCGAG | Error-prone PCR universal_Primer_R for full ORF subcloned into pYZ12s |
| Yfz_Oli243 | GCCGCTTGGGTTATTTTGAGATCT | Error-prone PCR Primer_F for Leu-regulatory domain (Leu430-Ala618, BglII_ScLEU4_XhoI) of ScLEU4 |
| Yfz_Oli345 | ATCATGGCTAGCGAATTTAAGTACAACG GTAAGGTC | NheI_*Ll_ilvD*_F |
| Yfz_Oli346 | ATACATCTCGAGTCATTACAAGTCGGTAA CACAACCTTCAG | XhoI_*Ll_ilvD*_R |
| Jla_oli234 | GATCGCTAGCCTGAGATCGTTATTGCAAA GC | NheI_ScILV6_F |
| Jla_oli235 | AATTCTCGAGACCAGGTGGTAGTTGGGA AATG | XhoI_ScILV6 _R |
| Jla_oli276 | GAATCGCTAGCGTTAAAGAGAGTATTAT TGCTCTTGCTGAGC | NheI_ScLEU4_F |
| Jla_oli276R | TCATTACTCGAGTGCAGAGCCAGATGCC GCAGCATTCTTA | XhoI_ScLEU4_R |
| Jla_oli280 | CAGAGCATTCTCTAGGTTCTGGTTCTACG CAAGCTGCTTCTTACATCC | LEU4$^{\Delta S547}$_site-directed mutagenesis Primer-F |
| Jla_oli281 | GGATGTAAGAAGCAGCTTGCGTAGAACC AGAACCTAGAGAATGCTCTG | LEU4$^{\Delta S547}$_site-directed mutagenesis Primer-R |

| | | |
|---|---|---|
| Yfz_KO71 | CCAGCGTATACAATCTCGATAGTTGGTTTCCCGTTCTTTCCACTCCCGTCtacgctgcaggtcgacaacc | ScGAL80_KO_F |
| Yfz_KO72 | GTTTTTATAACGTTCGCTGCACTGGGGGCCAAGCACAGGGCAAGATGCTTccactagtggatctgatatcacc | ScGAL80_KO_R |
| Yfz_KO187 | AGAAAAAAAAGGATTCTCACACTAGAAGTTTACTGTAGACTTTTTCCTTACAAAAAGACAAGGAACAATCtacgctgcaggtcgacaacc | ScLEU4_KO_F |
| Yfz_KO188 | AGGAAAGGAAGTAAATAAATAAGTATAGAAATAAATAGAAGCGAATAAGTCCTGAAATACAGAAAAGTTCctagtggatctgatatcacc | ScLEU4_KO_R |
| Yfz_KO189 | ACTACATGTTTTCGTTAGAATAAATCACCCTATAAACGCAAAATCAGCTAGAACCTTAGCATACTAAAACtacgctgcaggtcgacaacc | ScBAT1_KO_F |
| Yfz_KO190 | AACAGATCCTCTGAGAGGAATTCTCGTTTTTTTTTTTTGGGGGGGGAGGGGATGTTTACCTTCATTATCActagtggatctgatatcacc | ScBAT1_KO_R |
| Yfz_KO229 | TGTTTTCGGCTTATAAGGGTCTTCTCCTTAGGATAATACTATCGGCACATTATCATTTAGCCGCGTAGCCtacgctgcaggtcgacaacc | ScLEU9_KO_F |
| Yfz_KO230 | TTTTCTGTGCCATTTATAAATAAAAATACATATATATATAACATGAGTAATCATAAGCTACTCCTTTCTActagtggatctgatatcacc | ScLEU9_KO_R |
| Yfz_KO266 | ATTGTAGCGCCTGTAATCTTTAGTAACGGATTCTTGTATTTTTTTGTAAACAGCCAAGAAAAAAGTAGAGtacgctgcaggtcgacaacc | ScILV3_KO_F |

| | | |
|---|---|---|
| Yfz_KO267 | TGCGAACAAAAAAGATGATGGAAAAGG AGAATCTCTATATATATATTCATCGATTG GGGCCTATAATGCActagtggatctgatatcacc | ScILV3_KO_R |
| Yfz_KO278 | AAGCTCACTAGTAAAGGCGGGAAATAGA ACATTGAGAACGTATTTTGATAtacgctgcagg tcgacaacc | ScTMA29_KO_F |
| Yfz_KO279 | AAAGTCTTACATGTATAAAAAGTATACA GATTTACTTAGTTTAGCTAGGTctagtggatctg atatcacc | ScTMA29_KO_R |
| Jla_KO1 | TATTTTCTACTCATAACCTCACGCAAAAT AACACAGTCAAATCAATCAAAtacgctgcaggt cgacaacc | ScPDC1_KO_F |
| Jla_KO2 | TACATAAAAATGCTTATAAAACTTTAACT AATAATTAGAGATTAAATCGCccactagtggat ctgatatcacc | ScPDC1_KO_R |
| Jla_KO3 | CATAATCAATCTCAAAGAGAACAACACA ATACAATAACAAGAAGAACAAAtacgctgca ggtcgacaacc | ScPDC5_KO_F |
| Jla_KO4 | AAAGTAAAAAAATACACAAACGTTGAAT CATGAGTTTTATGTTAATTAGCccactagtggat ctgatatcacc | ScPDC5_KO_R |
| Jla_KO5 | AGTATAAATAAAAAAACCCACGTAATATA GCAAAAACATATTGCCAACAAAtacgctgcag gtcgacaacc | ScPDC6_KO_F |
| Jla_KO6 | TAAGTTTATTTATTTGCAACAATAATTCG TTTGAGTACACTACTAATGGCccactagtggatc tgatatcacc | ScPDC6_KO_R |

| | | |
|---|---|---|
| Jla_KO22 | AAAATTTTAGAAATTTAAGGGAAAGCATCTCCACGAGTTTTAAGAACGATtacgctgcaggtcgacaacc | ScBAT2_KO |
| Jla_KO23 | AGTTTTATTCTTTTTAACTTTTAATTACTTTACGTAGCAATAGCGATACTccactagtggatctgatatcacc | ScBAT2_KO |
| Sag_333 | CACCAGAACTTAGTTTCGACGG | TEF-ScILV2_ddPCR_F |
| Sag_334 | GGCAGGTGTGTTGCGG | TEF-ScILV2_ddPCR_R |
| Sag_341 | CCACTCGGCGTACAGCTC | ZeoR_ddPCR_F |
| Sag_342 | GACTTCGTGGAGGACGACTT | ZeoR_ddPCR_R |
| Sag_347 | ATCCTGCGCGTTGACATAA | chrXIV:222kb_ddPCR_F[Ref.27] |
| Sag_348 | AGATTCCGTTGCTGGCTATC | chrXIV:222kb_ddPCR_R[Ref.27] |
| Sag_393 | GATGTGAGCGTTTGAGTGGTCTTGC | Aro10_ddPCR_F |
| Sag_394 | AACCCTGGTGATGTTGTCGTTTGTG | Aro10_ddPCR_R |

**Supplementary Figure 1**

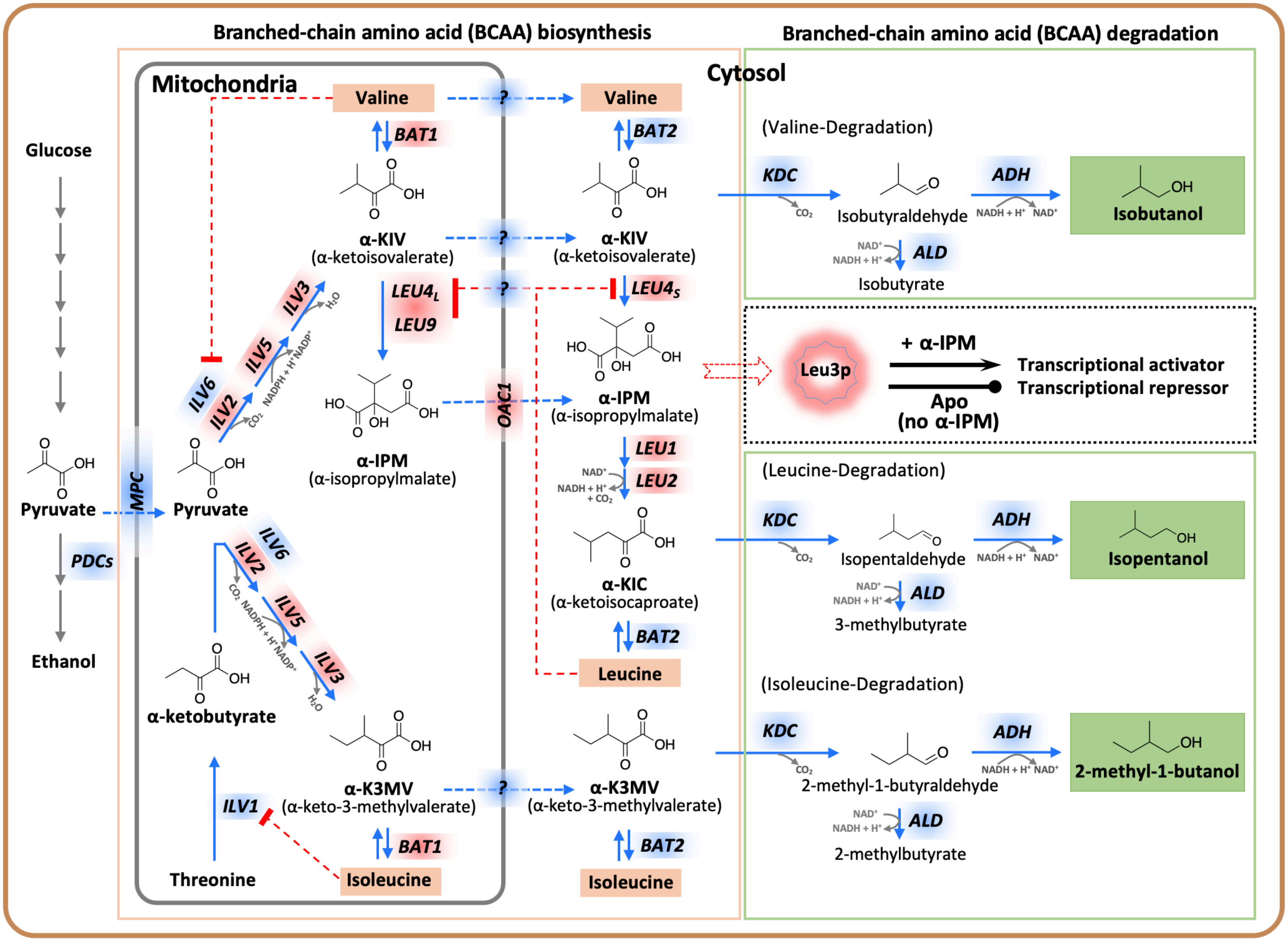

**Supplementary Figure 1. Pathways for branched-chain amino acid (BCAA) and branched-chain higher alcohol (BCHA) biosynthesis in *Saccharomyces cerevisiae.*** Isobutanol, isopentanol, and 2-methyl-1-butanol biosynthesis are derived from the biosynthesis and degradation of valine, leucine, and isoleucine, respectively. The upstream pathway for isobutanol production (valine biosynthesis) consists of three enzymes natively localized in mitochondria (orange rectangle): acetolactate synthase (ALS, encoded by *ILV2*), ketol-acid reductoisomerase (KARI, encoded by *ILV5*), and dehydroxyacid dehydratase (DHAD, encoded by *ILV3*)[28]. Ilv2p, Ilv3p, and Ilv5p convert two molecules of pyruvate to the valine precursor α-ketoisovalerate (α-KIV), which is exported to the cytosol by one or more unknown α-KIV carrier(s). The native localization of the Ehrlich valine degradation occurs in the cytosol, where α-KIV is converted to isobutanol through the Ehrlich BCAA degradation pathway[29] (green rectangle), comprised of α-ketoacid decarboxylases (α-KDCs) and alcohol dehydrogenases (ADHs). The conversion between α-KIV and valine is catalyzed by mitochondrial and cytosolic branched-chain amino acid aminotransferases (encoded by *BAT1* and *BAT2,* respectively). In the upstream pathway for isopentanol production (leucine biosynthesis), α-KIV is converted to α-isopropylmalate (α-IPM) by α-IPM synthases located in mitochondria (encoded by the short $LEU4_S$ and *LEU9*) and the cytosol (encoded by the long $LEU4_L$). Subsequently, α-IPM is converted in the cytosol to β-IPM by isopropylmalate isomerase (encoded by *LEU1*) and then to α-ketoisocaproate (α-KIC) by β-IPM dehydrogenase (encoded by *LEU2*). This α-KIC precursor is then converted to leucine by Bat2p. Alternatively, α-KIC is converted to isopentanol in the cytosol via the BCAA Ehrlich degradation pathway[29]. The upstream pathway for 2-methyl-1-butanol (isoleucine biosynthesis) consists of the same mitochondrial enzymes involved in valine and leucine biosynthesis, Ilv2p, Ilv5p, and Ilv3p; except that for isoleucine biosynthesis, Ilv2p catalyzes the condensation of one pyruvate and one α-ketobutyrate, produced by threonine deaminase (encoded by *ILV1*), instead of two pyruvate molecules as in the biosynthesis of valine and leucine. The subsequent reactions catalyzed by Ilv5p and Ilv3p result in the production of the isoleucine precursor α-keto-3-methylvalerate (α-K3MV), which is transaminated to isoleucine in the mitochondria and the cytosol by Bat1p and Bat2p, respectively. Alternatively, α-K3MV is converted to 2-methyl-1-butanol through the Ehrlich BCAA degradation pathway (green rectangle) in the cytosol. The enzymatic activities of Ilv6p, Leu4p/Leu9p, and Ilv1p are negatively regulated by valine, leucine, and isoleucine, respectively, as indicated with red dashed lines. Leu3p, a dual-function transcriptional regulator, regulates genes (highlighted in red) involved in BCAA biosynthesis. It acts as a transcriptional activator in the presence of α-isopropylmalate (α-IPM) and a repressor in its absence. Genes not known to be regulated by Leu3p are labeled in blue. The blue rectangle denotes the cell plasma membrane. *PDC*s: pyruvate decarboxylase isozymes; *MPC*: mitochondrial pyruvate carriers; *OAC1*: mitochondrial α-IPM transporter; *ALD*: aldehyde dehydrogenase.

**Supplementary Figure 2**

**a)** ***leu2Δ* strain for isobutanol production and biosensor configuration**

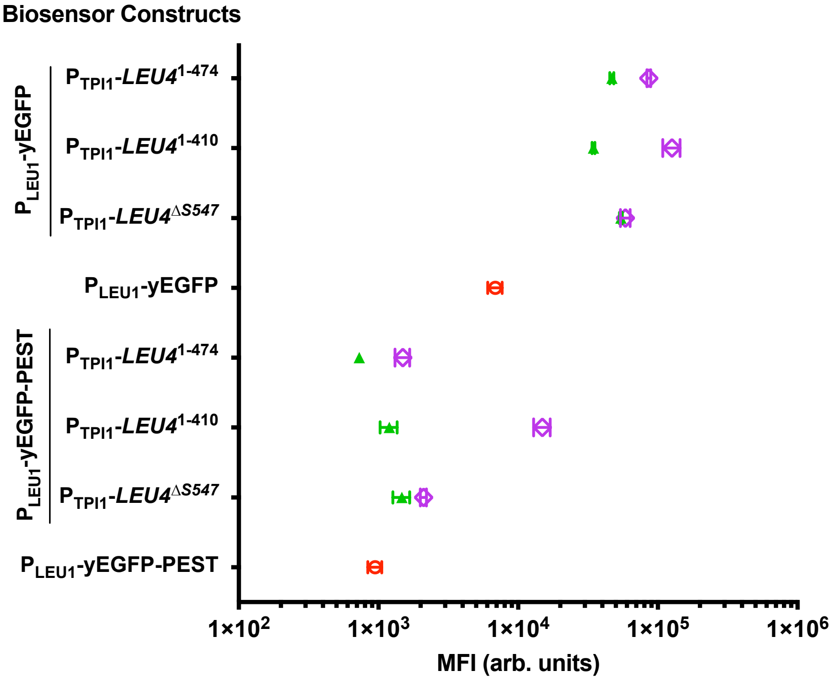


**b)** ***LEU2* strain for isopentanol production and biosensor configuration**

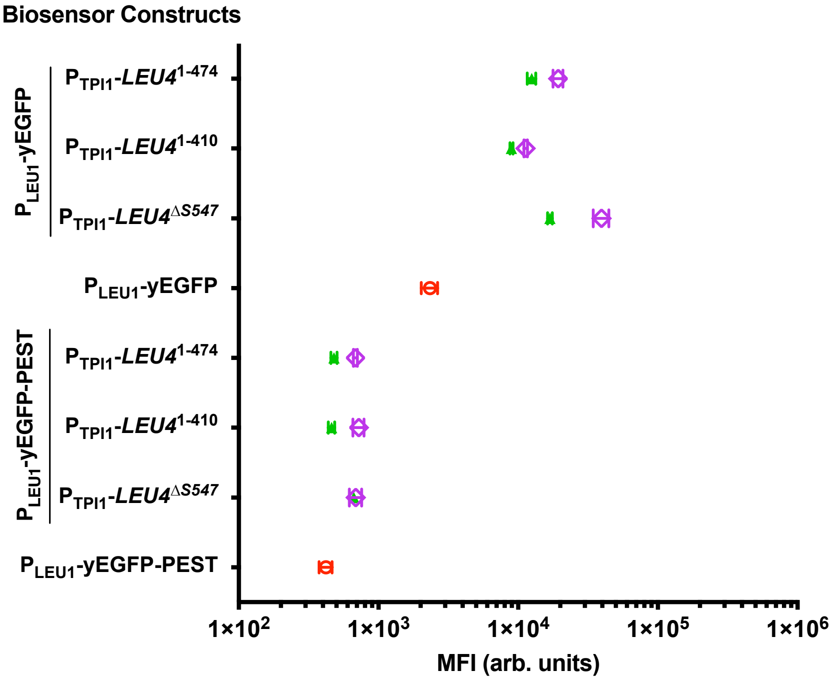

**Supplementary Figure 2. GFP median fluorescence intensity (MFI) of different biosensor constructs.** Biosensor MFI was measured in a *leu2*Δ strain (a) to develop a biosensor configuration specific to isobutanol and in a *LEU2* strain (b) to develop a biosensor configuration specific to isopentanol. The BCHA biosensor is off (red circles) when only $P_{LEU1}$-yEGFP-PEST-$T_{ADH1}$ or $P_{LEU1}$-yEGFP-$T_{ADH1}$ are introduced because the endogenous α-IPM synthase (Leu4p) is inhibited by leucine. The BCHA biosensor constructs can be turned on (green triangles) when a leucine-insensitive Leu4 variant ($LEU4^{\Delta S547}$, $LEU4^{1\text{-}410}$, or $LEU4^{1\text{-}474}$) is additionally introduced. For each Leu4p mutant, the MFI is reported for constructs with and without a PEST tag fused to yEGFP. The MFI increases when the BCHA biosynthesis pathway (*ILV2, ILV3, ILV5*, KDC, and ADH) is overexpressed in strains harboring different biosensor constructs (purple diamonds). MFI are represented in arbitrary units (arb. units). All data are shown as mean values. Error bars represent the standard deviation of at least three biological replicates. Source data are provided as a Source Data file.

**Supplementary Figure 3**

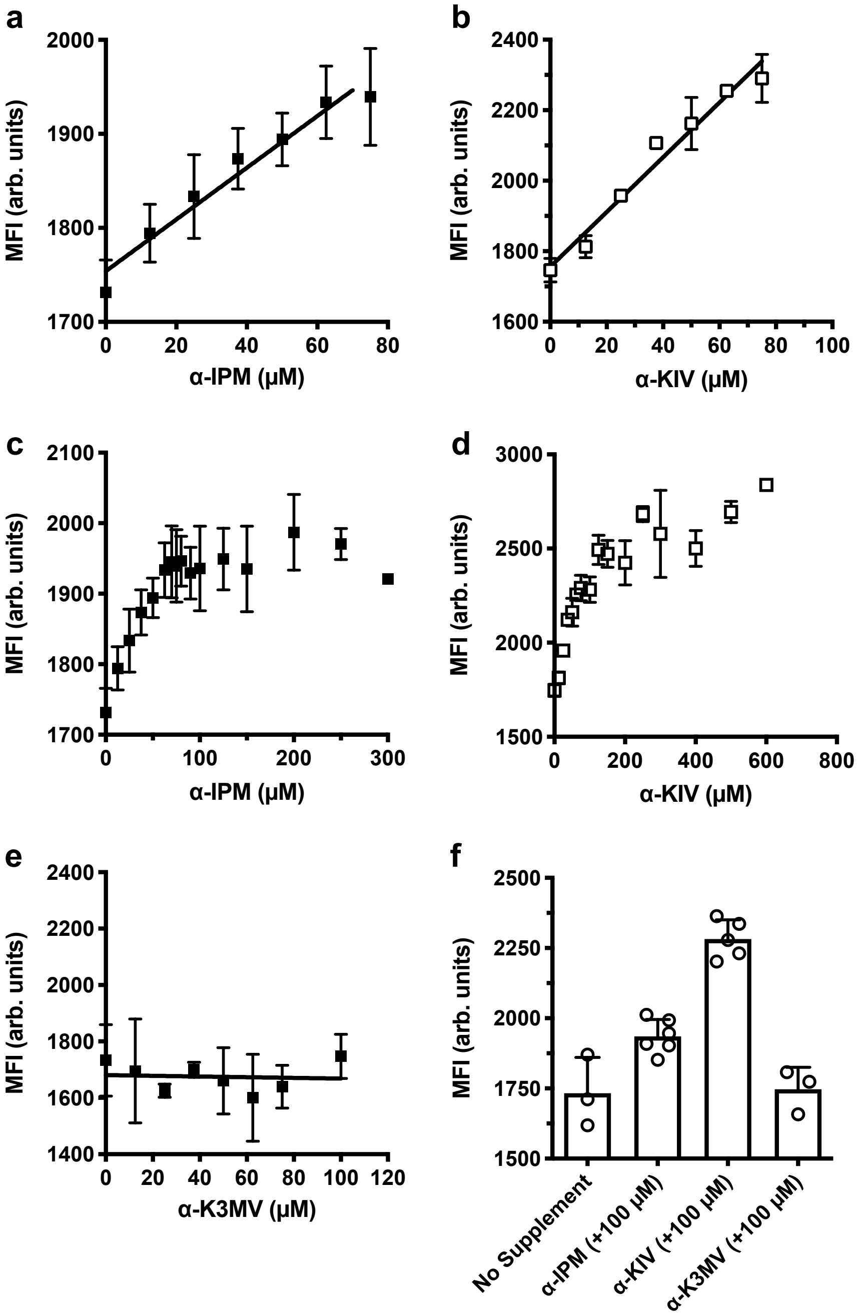


**Supplementary Figure 3. Response of the biosensor in its isobutanol configuration to isopropylmalate (α-IPM), α-ketoisovalerate (α-KIV), and α-keto-3-methylvalerate (α-K3MV).** The biosensor responds linearly to increasing concentrations of α-IPM (**a**) and α-KIV (**b**) supplemented in the media up to 80 μM. Biosensor response to higher concentrations of **(c)** α-IPM, **(d)** α-KIV, or **(e)** α-K3MV supplemented in the media. **f)** Side-by-side comparison of the biosensor response to 100 μM of α-IPM, α-KIV, and α-K3MV supplemented in the media. Median fluorescence intensity (MFI) are represented in arbitrary units (arb. units). All data are shown as mean values. Error bars represent the standard deviation of at least three biological replicates. Source data are provided as a Source Data file.

**Supplementary Figure 4**

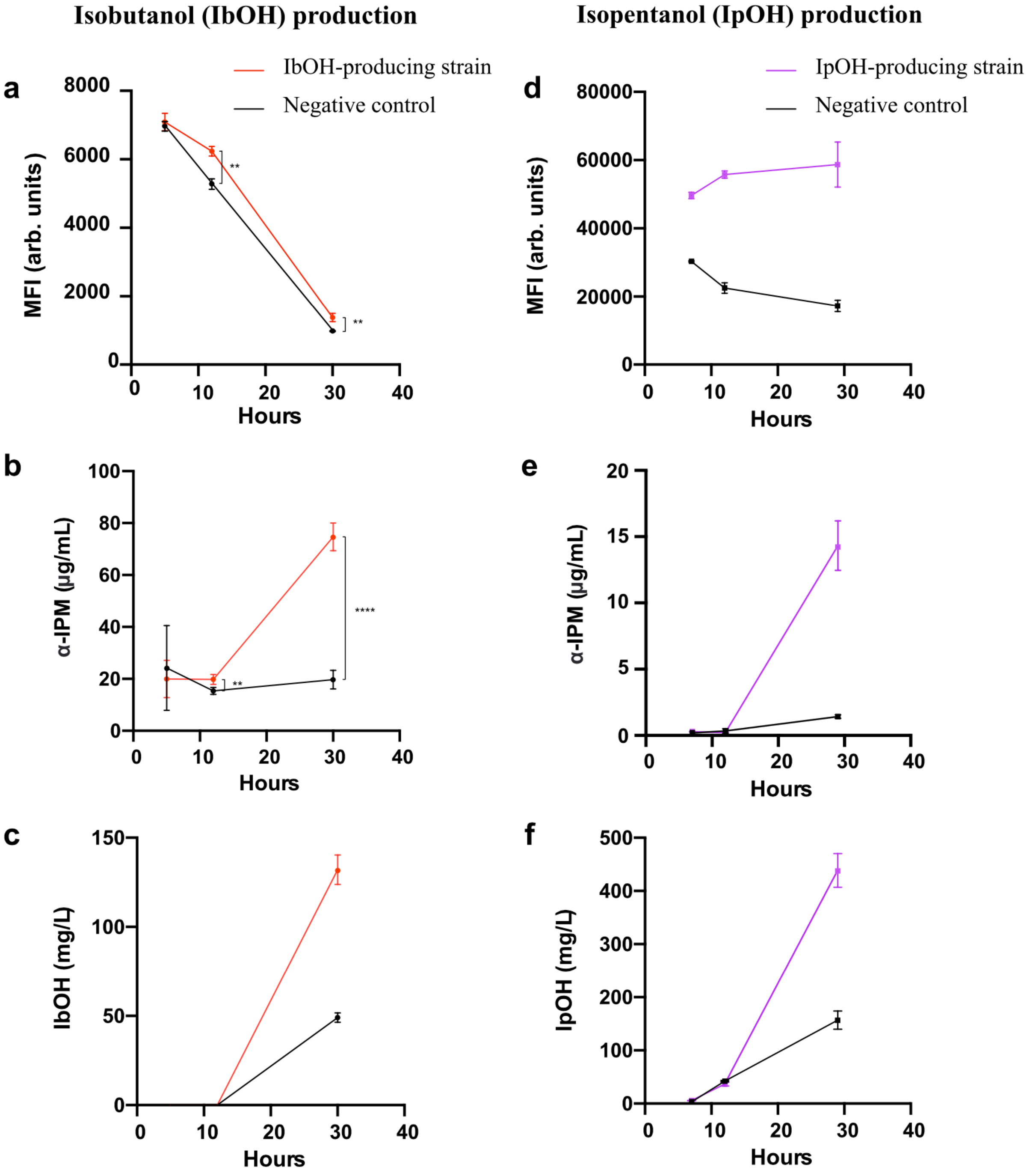


**Supplementary Figure 4. Correlations of biosensor response to intracellular alpha-isopropylmalate (α-IPM) concentrations, and BCHA production in low-cell density fermentations. (a)** Median fluorescent intensity (MFI) measured throughout a low-cell density fermentation with an isobutanol-producing strain (red, YZy235) and a negative control strain (black, YZy121), both carrying the isobutanol-configured biosensor. **(b)** Intracellular α-IPM

concentrations measured during the low-cell-density fermentations for both the isobutanol producer and control strains. A two-sided *t*-test was used to determine the statistical significance of the differences in MFI measurements and α-IPM concentrations in the low- and high-producers; From left to right in Supplementary Figure 4a: $P$=0.0014, $P$=0.0046; From left to right in Supplementary Figure 4b: $P$=0.001, $P$<0.000001; ** $P \leq 0.01$ **** $P \leq 0.0001$. **(c)** Isobutanol production of the isobutanol-producer and control strains. No detectable measurements at 5h and 12h. **(d)** MFI measured throughout a low-cell-density isopentanol fermentation in an isopentanol-producing strain (purple, SHy159) and a negative control strain (black, SHy187), both containing the isopentanol-configured biosensor. **(e)** Intracellular α-IPM concentrations measured during the low-cell-density fermentations for both the isopentanol producer and control strains. **(f)** Isopentanol production of isopentanol-producer and control strains. MFI are represented in arbitrary units (arb. units). All data are shown as mean values. Error bars represent the standard deviation of at least three biological replicates. Source data are provided as a Source Data file.

**Supplementary Figure 5**

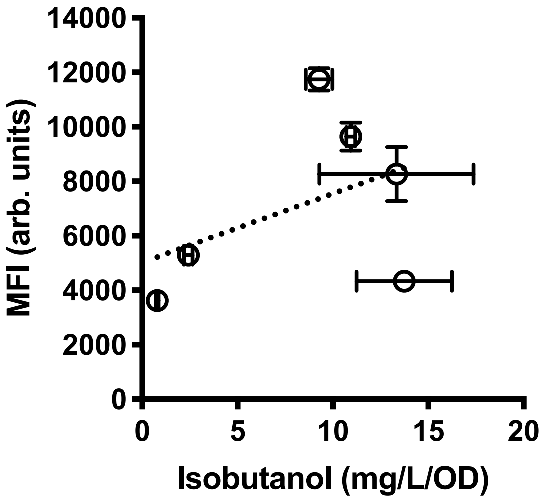


**Supplementary Figure 5. Correlation between specific isobutanol titers and GFP fluorescence signals from the isopentanol configuration of the biosensor in the *LEU2* strains engineered for isopentanol production.** Strains used from left to right: SHy187, SHy192, SHy158, SHy176, SHy159, and SHy188 (Supplementary Table 1). The median fluorescence intensity (MFI) for each strain was measured after 13h of growth and plotted with the corresponding specific isobutanol titers obtained after 48h high-cell-density fermentations. The dotted line shows a poor linear regression fit with an $R^2$ of 0.19. MFI are represented in arbitrary units (arb. units). All data are shown as mean values. Error bars represent the standard deviation of at least three biological replicates. Source data are provided as a Source Data file.

## Supplementary Figure 6

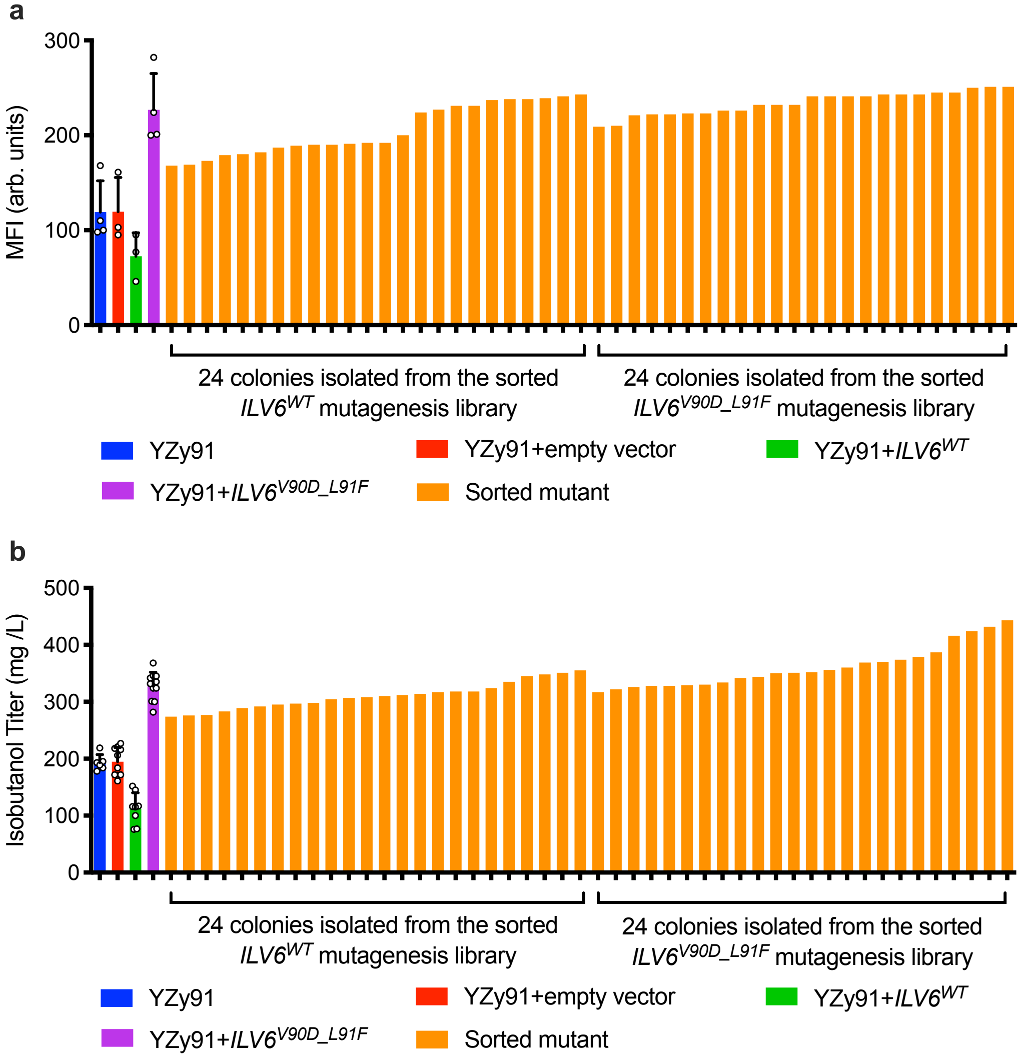


**Supplementary Figure 6. Screen of 48 random colonies sorted from mutagenesis libraries of the wild-type *ILV6* and valine-insensitive *ILV6*$^{V90D\_L91F}$ mutant.** (**a**) Flow cytometry measurements of the GFP median fluorescence intensity (MFI) of 48 sorted strains (orange) measured after 13h of growth in media containing four times more valine (2.4 mM) than the usual synthetic defined medium (24 colonies were isolated from the sorted wild-type *ILV6* mutagenesis library, and 24 colonies were isolated from the sorted *ILV6*$^{V90D\_L91F}$ mutagenesis library). MFI of the basal strain YZy91 (*ilv6Δ bat1Δ bat2Δ*) with (red) or without (blue) an empty vector, or transformed with plasmids containing *ILV6*$^{WT}$ (green) or *ILV6*$^{V90D\_L91F}$ (purple), are shown as controls. (**b**) Isobutanol titers after 48h fermentations with the same 48 sorted strains (orange) and controls (red, blue, purple, and green) shown in (**a**). MFI are represented in arbitrary units (arb. units). All data except the data of sorted mutant are shown as mean values. Open circles represent individual data points. Error bars of the control strains represent the standard deviation of at least three biological replicates. Source data are provided as a Source Data file.

**Supplementary Figure 7**

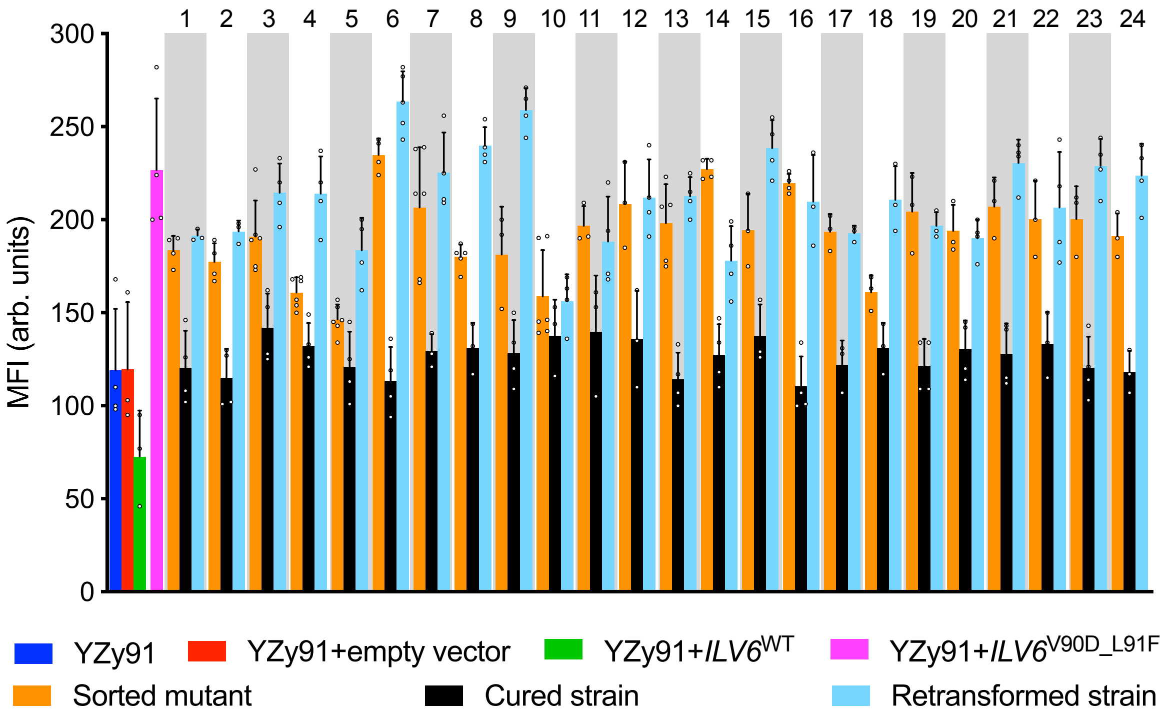


**Supplementary Figure 7. Confirmation that isolated *ILV6* variants with unique sequences enhance GFP fluorescence signal from the isobutanol-configured biosensor.** Flow cytometry measurements of the GFP median fluorescence intensity (MFI) of sorted strains with unique *ILV6* sequences (orange), plasmid-cured derivatives (black), and strains obtained from retransforming YZy91 with each unique plasmid isolated from the corresponding sorted strains (cyan), measured after 13h of growth in media containing four times more valine (2.4 mM) than the usual synthetic defined medium. GFP fluorescence measured with the basal strain YZy91 (*ilv6Δ bat1Δ bat2Δ*) with (red) or without (blue) an empty vector, or transformed with plasmids containing wild-type *ILV6* (*ILV6*$^{WT}$, green), or *ILV6*$^{\mathrm{V90D\_L91F}}$ (magenta), are shown as controls. The numbers on the upper x-axis identify each of the strains harboring screened mutants with unique *ILV6* sequences. *ILV6* mutants 1 – 10 were isolated from the sorted *ILV6*$^{\mathrm{WT}}$ mutagenesis library. *ILV6* mutants 11 – 24 were isolated from the sorted *ILV6*$^{\mathrm{V90D\_L91F}}$ mutagenesis library. MFI are represented in arbitrary units (arb. units). All data are shown as mean values. Open circles represent individual data points. Error bars represent the standard deviation of three biological replicates. Source data are provided as a Source Data file.

**Supplementary Figure 8**

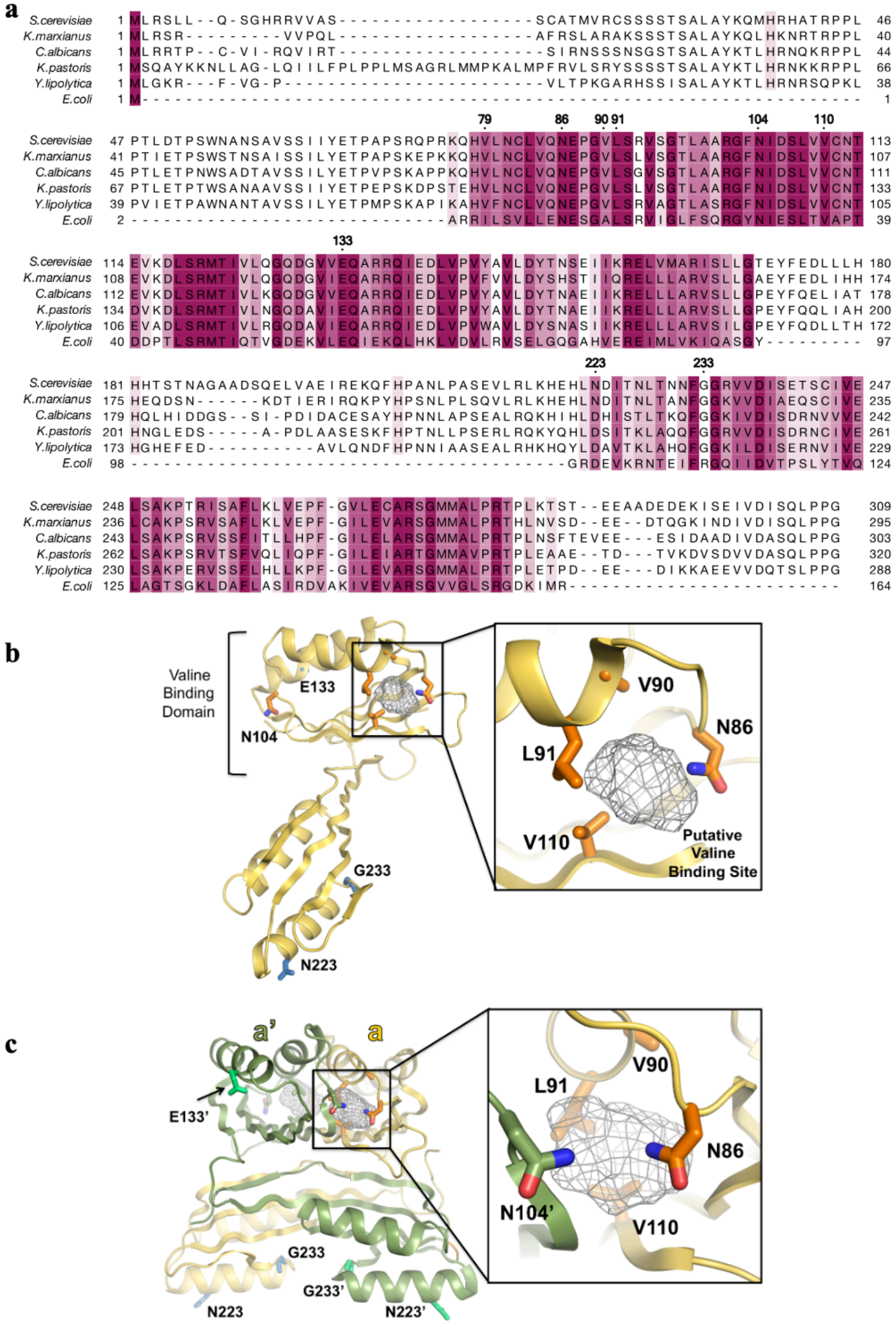

**Supplementary Figure 8. Sequence and structural analysis of yeast Ilv6p and its isolated mutants.** (**a**) Alignment of the amino acid sequence of *E.coli* IlvH (acetohydroxyacid synthase regulatory subunit) with Ilv6p sequences from *Saccharomyces cerevisiae*, *Kluyveromyces marxianus*, *Candida albicans*, *Komagataella pastoris*, and *Yarrowia lipolytica*. The color intensity reflects the level of conservation of each residue. Key residues mutated in *ILV6*, derived from the *ILV6* wild-type mutagenesis library (Supplementary Table 3), that enhance isobutanol production and whose positions are present in *E.coli* IlvH (N86, V90, L91, N104, V110, N223, E133, and G233) are labeled on the top of the alignment (most of them are highly conserved except G233). (**b, c**) Key substituted residues above mapped onto the crystal structure of the regulatory subunit of acetohydroxyacid synthase, IlvH, from *E. coli* (pdb code: 2f1f), shown on the structure of the monomer (**b**), and from a different angle on the structure of the dimer (**c**). Close-up views of the putative valine-binding sites (gray mesh) and the key residues that make them are shown in the side-boxes.

## Supplementary Figure 9

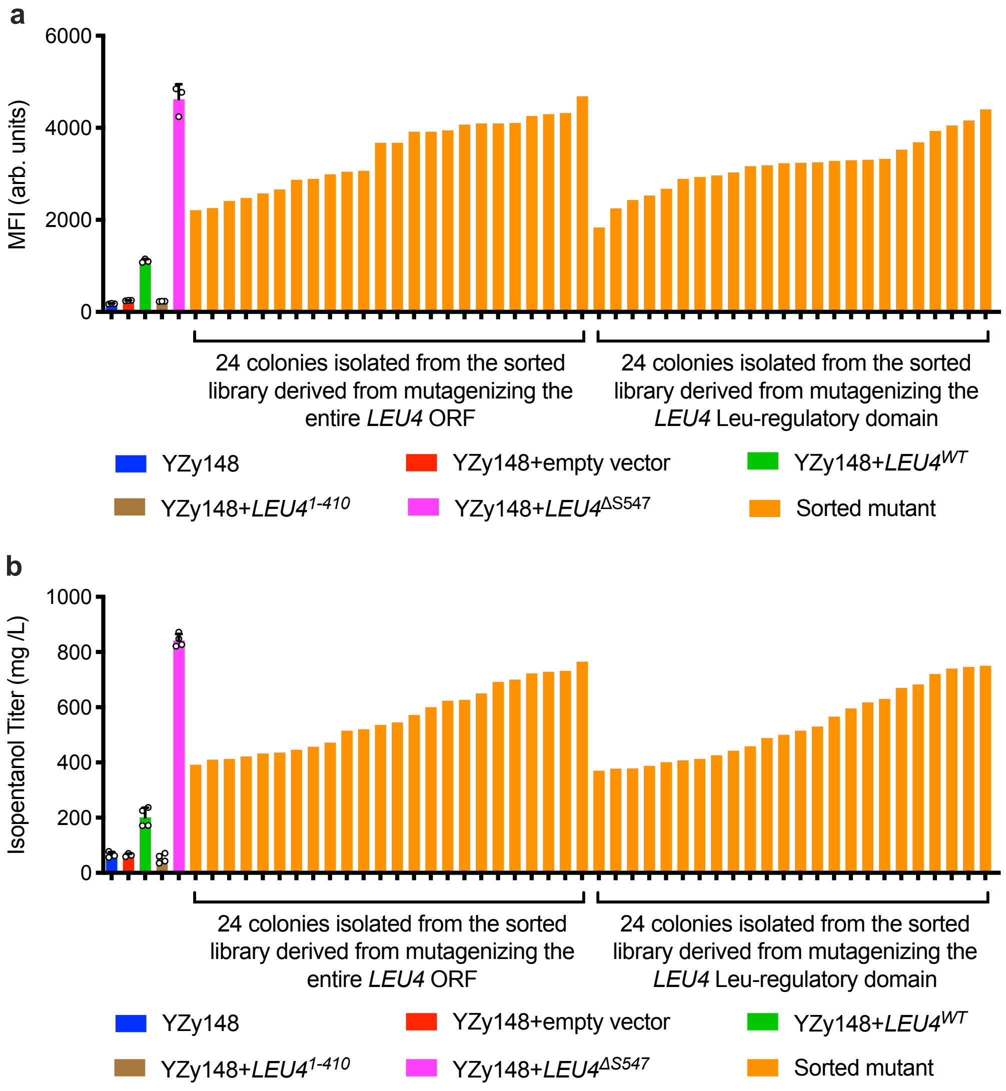


**Supplementary Figure 9. Screen of 48 random colonies sorted from libraries derived from mutagenizing the entire *LEU4* ORF or the *LEU4* regulatory domain.** (**a**) Flow cytometry measurements of the GFP median fluorescence intensity (MFI) of 48 sorted strains (orange) measured after 13 h of growth in SC-Ura media supplemented with 2% glucose (24 colonies are isolated from the sorted library derived from mutagenizing the entire *LEU4* ORF, and 24 colonies are isolated from the sorted library derived from mutagenizing the *LEU4* regulatory domain). MFI of the basal strain YZy148 (*leu4Δ leu9Δ bat1Δ LEU2*) with (red) or without (blue) an empty vector, or transformed with plasmids containing wild-type *LEU4* ($LEU4^{WT}$, green), $LEU4^{1-410}$ (brown), or $LEU4^{\Delta S547}$ (magenta), are shown as controls. (**b**) Isopentanol titers after 48h fermentations with the same 48 sorted strains (orange) and controls (red, blue, green, brown, and magenta) shown in (**a**). MFI are represented in arbitrary units (arb. units). All data except the data of sorted mutant are shown as mean values. Open circles represent individual data points. Error bars of the control strains represent the standard deviation of at least three biological replicates. Source data are provided as a Source Data file.

## Supplementary Figure 10

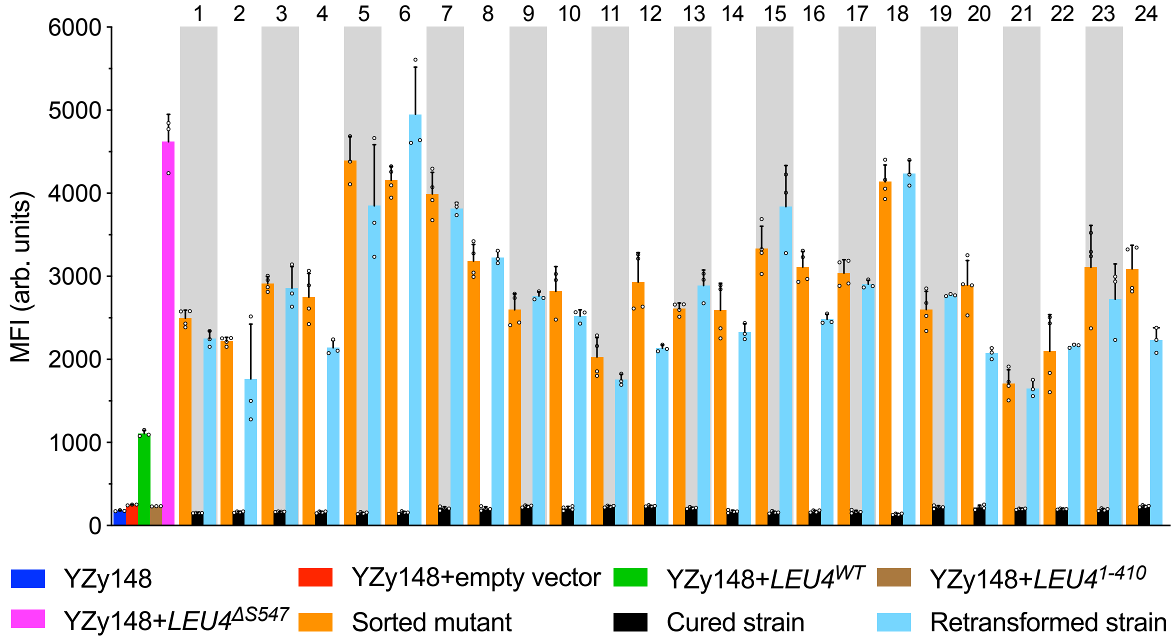


**Supplementary Figure 10. Confirmation that isolated *LEU4* variants with unique sequences enhance GFP fluorescence signal from the isopentanol-configured biosensor.** Flow cytometry measurements of the GFP median fluorescence intensity (MFI) of sorted strains with unique *LEU4* sequences (orange), plasmid-cured derivatives (black), and strains obtained from retransforming YZy148 with each unique plasmid isolated from the corresponding sorted strains (cyan), measured after 13 h of growth in SC-Ura media supplemented with 2% glucose. GFP fluorescence intensity of the basal strain YZy148 (*leu4Δ leu9Δ bat1Δ LEU2*) with (red) or without (blue) an empty vector, or transformed with plasmids containing wild-type *LEU4* (*LEU4*$^{WT}$, green), *LEU4*$^{1\text{-}410}$ (brown), or *LEU4*$^{\Delta S547}$ (magenta), are shown as controls. The numbers on the upper x-axis identify each of the strains harboring screened mutants with unique *LEU4* sequences. *LEU4* mutants 1–13 were isolated from the sorted library derived from mutagenizing the entire *LEU4* ORF. *LEU4* mutants 14–24 were isolated from the sorted library derived from mutagenizing the *LEU4* leucine regulatory domain. MFI are represented in arbitrary units (arb. units). All data are shown as mean values. Open circles represent individual data points. Error bars represent the standard deviation of three biological replicates. Source data are provided as a Source Data file.

## Supplementary Figure 11

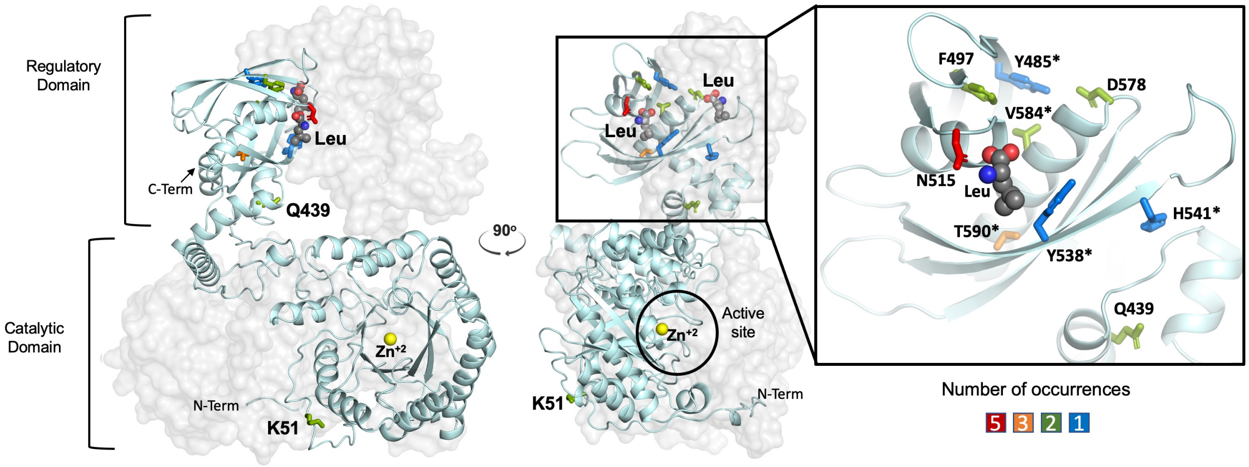


**Supplementary Figure 11. Structural analysis of key residues mutated in isolated Leu4p variants.** Key residues found to be mutated in Leu4p variants that enhance isopentanol production mapped onto the crystal structure of LeuA from *Mycobacterium tuberculosis* (pdb code: 3fig). The catalytic and regulatory domains of the LeuA dimer are depicted (left panel). One of the two monomers is represented as a cartoon (light blue) and the other one as a surface (gray). A close-up view of the occupied leucine binding site of the regulatory domain (right panel box) shows the orientation of key residues (shown in sticks) found to be mutated in isolated *LEU4* variants that enhance isopentanol production. Residues labeled with an asterisk (*) are those mutated in variants containing only one mutation (see Supplementary Table 5). The number of unique sequences in which each residue is found mutated is depicted by a color scale shown in the bottom right corner (see Supplementary Tables 4, 5). Leucine bound to the regulatory binding site and a zinc ion are show in spheres.

**Supplementary Figure 12**

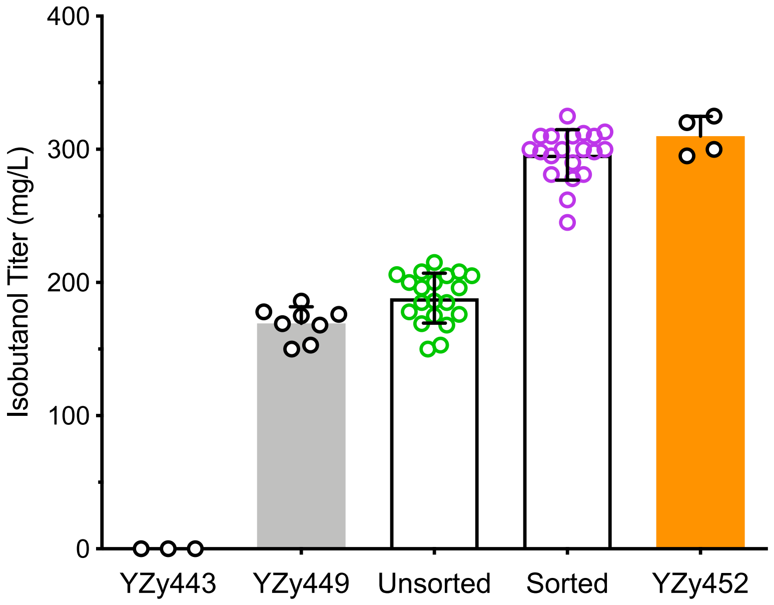


**Supplementary Figure 12. High-throughput screens, enabled by the isobutanol configuration of the biosensor, to identify strains carrying extra copies of *Ec_ilvC*$^{P2D1\text{-}A1}$ with enhanced isobutanol production from galactose.** Isobutanol production of the parent strain (YZy443), the baseline strain containing a single copy of a genomically integrated galactose-inducible isobutanol cytosolic pathway (YZy449), 20 random colonies from each the unsorted population (unsorted) and sorted population (sorted) of YZy449 transformed with extra copies of *Ec_ilvC*$^{P2D1\text{-}A1}$ randomly integrated into δ-sites, and the best sorted strain (YZy452). Measurements were made after 48h fermentations in 15% galactose. All data are shown as mean values. Open circles represent individual data points. Error bars represent the standard deviation of at least three biological replicates (for YZy443, YZy449 and YZy452) or 20 random colonies (from unsorted and sorted populations). Source data are provided as a Source Data file.

**Supplementary Figure 13**

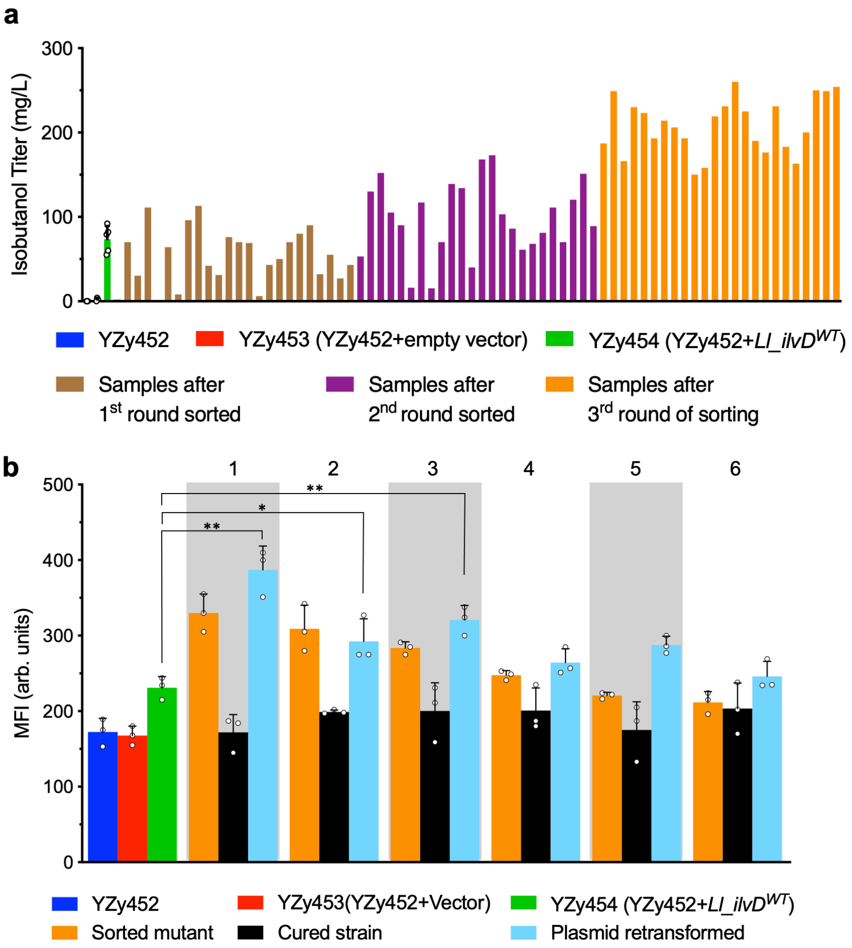


**Supplementary Figure 13. High-throughput screens for *Ll_ilvD* variants with enhanced cytosolic isobutanol production from glucose using the isobutanol configuration of the biosensor.** (**a**) Isobutanol titers of 24 colonies randomly picked after each round of FACS. Titers obtained with the basal strain YZy452 (containing the cytosolic isobutanol pathway with extra copies of *Ec_ilvC*$^{P2D1\text{-}A1}$ and galactose-inducible *Ll_ilvD*) with (red) or without (blue) an empty vector, or transformed with a plasmid containing wild-type *Ll_ilvD* (*Ll_ilvD*$^{WT}$, green) are shown as controls. (**b**) Confirmation that isolated *Ll_ilvD* variants with unique sequences enhance GFP fluorescence signal from the isobutanol-configured biosensor. Flow cytometry measurements of the GFP median fluorescence intensity (MFI), taken after 13h of growth in 2% glucose, for sorted strains (orange), plasmid-cured derivatives (black), and basal strain (YZy452) retransformed with each unique plasmid isolated from the corresponding sorted strains (cyan). Fluorescence of the basal strain YZy452 with (red) or without (blue) an empty vector, or transformed with a plasmid containing *Ll_ilvD*$^{WT}$ (green) are shown as controls. The numbers on the upper x-axis identify each of the strains harboring unique *Ll_ilvD* variants. MFI are represented in arbitrary units (arb. units). Except the data of 24 colonies randomly picked after each round of FACS, all data are shown as mean values. Open circles represent individual data points. Error bars represent the standard deviation of three biological replicates. A two-sided *t*-test was used to determine the statistical significance of the difference between MFI of YZy452 transformed with a plasmid containing

*Ll_ilvD*$^{WT}$ (green), or *Ll_ilvD* mutant #1 (*Ll_ilvD*$^{I433V}$), or mutant #2 (*Ll_ilvD*$^{V12A,\ S189P,\ H439R}$), or mutant #3 (*Ll_ilvD*$^{K535R}$). From left to right: $P$=0.0015, $P$=0.0336, $P$=0.003; * $P \leq 0.05$, ** $P \leq 0.01$. Source data are provided as a Source Data file.

**Supplementary Figure 14**

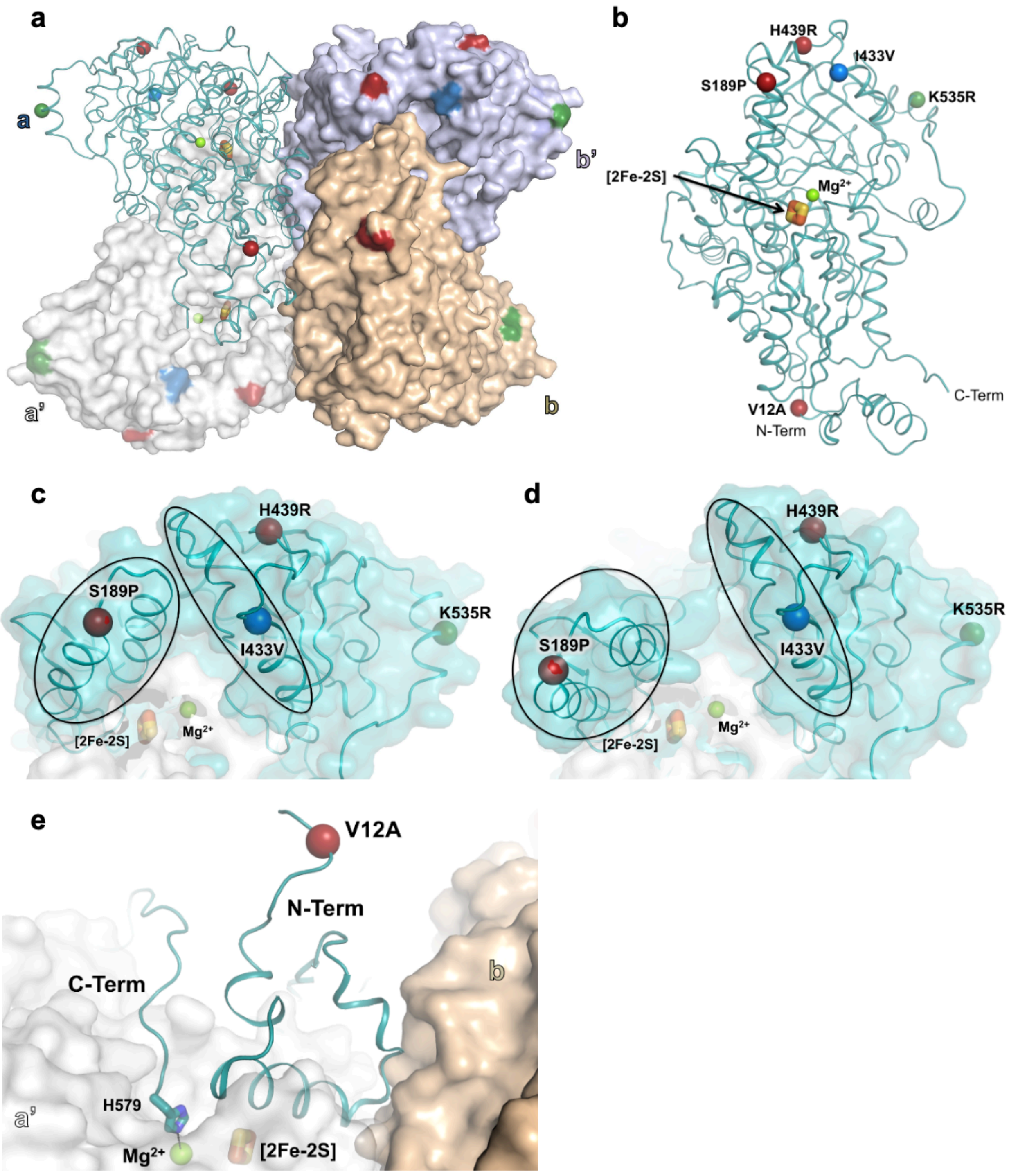


**Supplementary Figure 14. Structural analysis of key residues mutated in isolated IlvD variants.** Key residues found to be mutated in *Ll*_IlvD variants that enhance cytosolic isobutanol production mapped onto the crystal structure of its homolog l-arabinonate dehydratase from *Rhizobium leguminosarum bv. trifolii* (pdb code: 5j84). The colored spheres indicate the locations

of the mutated residues found in *Ll*_IlvD mutants #1 (blue), #2 (red) and #3 (green), (see Supplementary Table 8) (**a**) Tetramer of l-arabinonate dehydratase, a homolog of *Ll*_IlvD with 31.56% sequence identity and 36% sequence similarity. Residues substituted in *Ll*_IlvD mutants #1 (blue), #2 (red), and #3 (green) are shown as spheres on one of the four monomers represented as a ribbon (light blue), and colored on the surface representations (light blue, wheat, gray) of the other three monomers. (**b**) Ribbon representation of an *Ll*_IlvD monomer showing the positions of mutated residues (spheres) found in *Ll*_IlvD mutants #1 (blue), #2 (red), and #3 (green), relative to the 2Fe-2S cluster (orange and yellow sticks) and the $Mg^{2+}$ ion (bright green sphere) bound to the active site. (**c, d**) Close-up views of the closed (**c**) and open (**d**) conformations of the enzyme (pdb code: 5j84 and pdb code: 5j85, respectively). Residues S189 (substituted in mutant #2) and I433 (substituted in mutant #1), are located in the lobes (circled in black) that open and close to grant access or protect to the active site. (**e**) A close-up view shows the packing between the N- and C-termini of a monomer, which potentially contributes to the positioning of the His-579 that coordinates the $Mg^{2+}$ in the active site (found in a different monomer).

**Supplementary Figure 15**

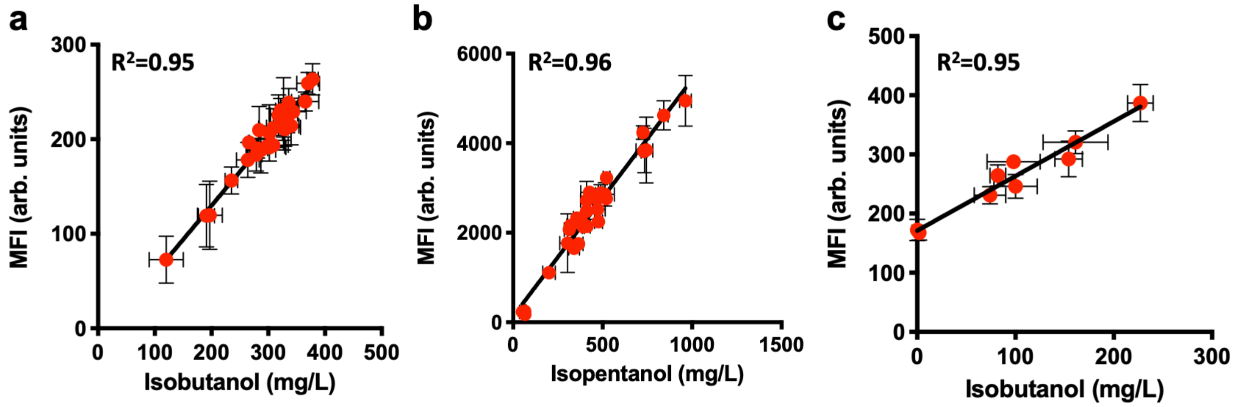


**Supplementary Figure 15. Correlation between BCHAs titers and GFP fluorescence signals from the isobutanol or isopentanol configurations of the biosensor in strains screened during three applications. (a)** The strains obtained by retransforming YZy91 with an empty vector, or plasmids containing *ILV6$^{WT}$*, *ILV6$^{V90D_L91F}$*, or each unique *ILV6* variant isolated from three rounds of FACS of *ILV6* mutagenized libraries **(**Fig. 3b**)** using the butanol configuration of the biosensor**. (b)** The strains obtained by retransforming YZy148 with an empty vector, or plasmids containing *LEU4$^{WT}$* or each unique *LEU4* variant isolated from three rounds of FACS of *LEU4* mutagenized libraries **(**Fig. 4c**)** using the isopentanol configuration of the biosensor. **(c)** The strains obtained by retransforming YZy452 with an empty vector, or plasmids containing *Ll_ilvD$^{WT}$*, or each unique *Ll_ilvD* variant isolated from three rounds of FACS of *Ll_ilvD* mutagenized libraries (Fig. 5b**)** using the butanol configuration of the biosensor. The GFP median fluorescence intensity (MFI) for each strain was measured after 13h of growth and plotted with the corresponding BCHA titers obtained after 48h high-cell-density fermentations. The solid lines show strong linear regression fits with $R^2$ values of 0.95, 0.96, 0.95 in figures (a), (b) and (c), respectively. MFI are represented in arbitrary units (arb. units). All data are shown as mean values. Error bars represent the standard deviation of at least three biological replicates. Source data are provided as a Source Data file.

**Supplementary Figure 16**

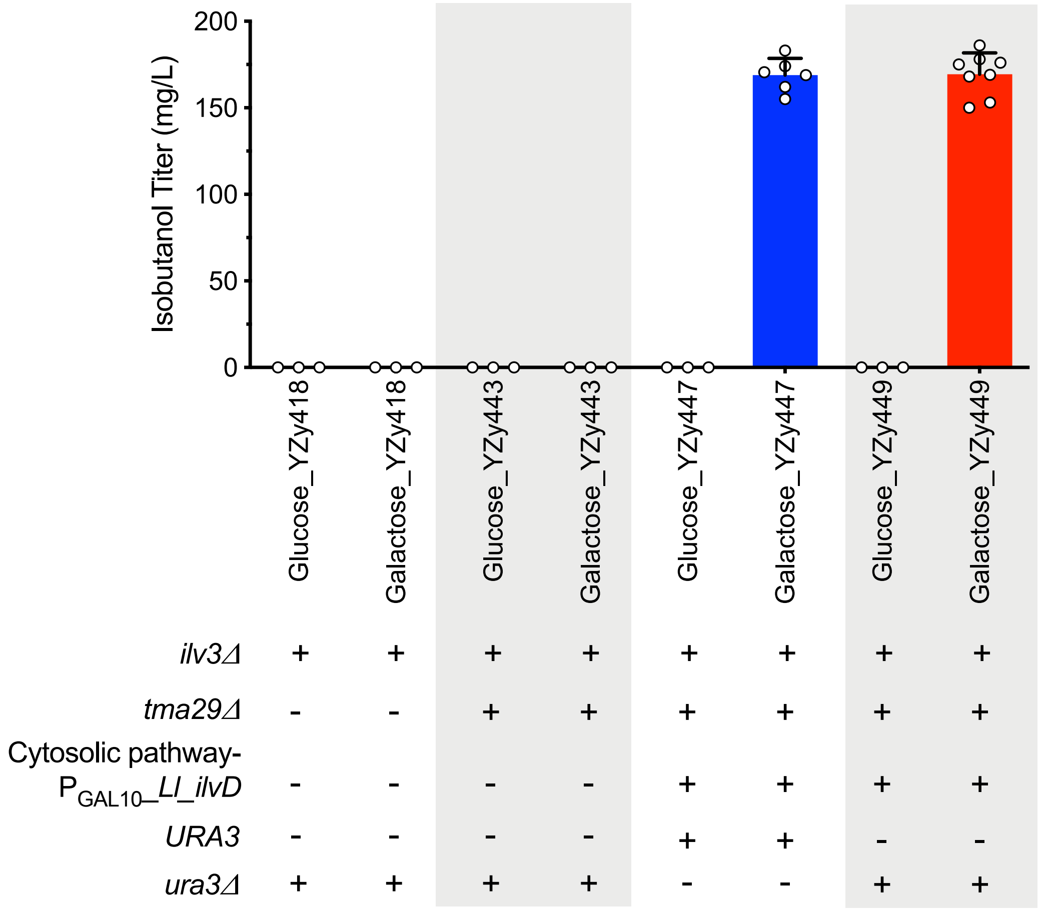


**Supplementary Figure 16. Isobutanol production from glucose and galactose in engineered strains with or without galactose-inducible *Ll_ilvD*.** Strains were fermented for 48h using 15% glucose or galactose as the carbon source. YZy418 and YZy443 produced no detectable isobutanol from either carbon source. All data are shown as mean values. Open circles represent individual data points. Error bars represent the standard deviation of at least three biological replicates. Source data are provided as a Source Data file.

**Supplementary Figure 17**

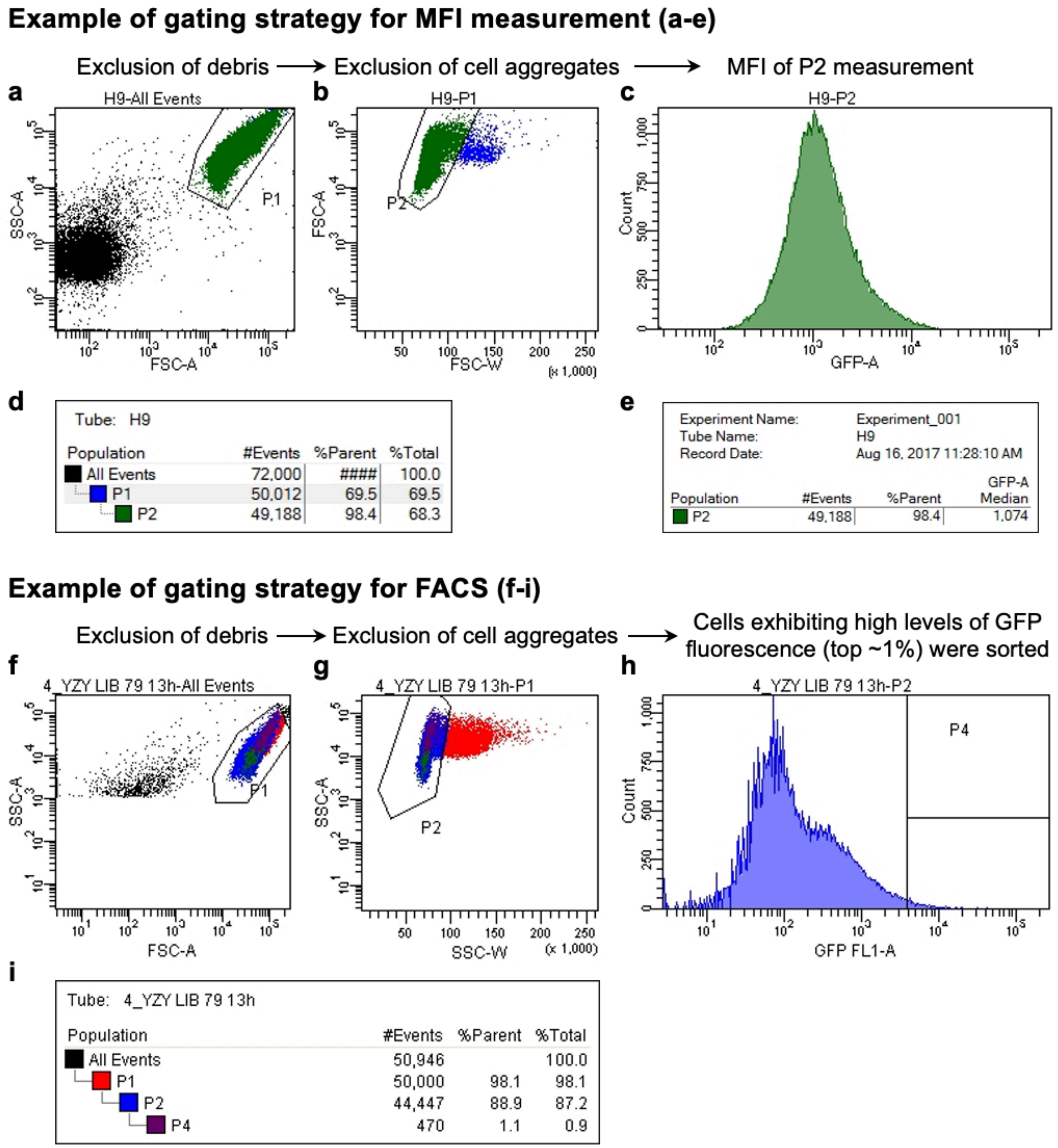


**Supplementary Figure 17. Flow cytometry gating strategies for GFP median fluorescence intensity (MFI) measurement (a-e) and fluorescence activated cell sorting (FACS) (f-i).** Cells were gated on forward scatter (FSC) and side scatter (SSC) signals to discard debris (**a** and **f**). We next plotted the FSC-width (FSC-W) against the FSC-area (FSC-A) (alternatively, SSC-W vs. SSC-A can also be used) to exclude the doublets and multiplets (**b** and **g).** After eliminating debris and cell aggregates, we measured the MFI of P2 (**c**). Cells exhibiting high levels of GFP fluorescence (top ~1%, P4) were sorted (**h**). The statistical tables are show in **d**, **e**, and **i**.

## Supplementary References


1 Sze, J. Y., Woontner, M., Jaehning, J. A. & Kohlhaw, G. B. In vitro transcriptional activation by a metabolic intermediate: activation by Leu3 depends on alpha-isopropylmalate. *Science* **258**, 1143-1145, doi:10.1126/science.1439822 (1992).

2 Zhou, K. M., Bai, Y. L. & Kohlhaw, G. B. Yeast regulatory protein LEU3: a structure-function analysis. *Nucleic Acids Res* **18**, 291-298, doi:10.1093/nar/18.2.291 (1990).

3 Cavalieri, D. *et al.* Trifluoroleucine resistance and regulation of alpha-isopropyl malate synthase in Saccharomyces cerevisiae. *Mol Gen Genet* **261**, 152-160, doi:10.1007/s004380050952 (1999).

4 Hammer, S. K., Zhang, Y. & Avalos, J. L. Mitochondrial compartmentalization confers specificity to the 2-ketoacid recursive pathway: increasing isopentanol production in Saccharomyces cerevisiae. *ACS Synth Biol* **9**, 546-555, doi:10.1021/acssynbio.9b00420 (2020).

5 Mateus, C. & Avery, S. V. Destabilized green fluorescent protein for monitoring dynamic changes in yeast gene expression with flow cytometry. *Yeast* **16**, 1313-1323, doi:10.1002/1097-0061(200010)16:14<1313::AID-YEA626>3.0.CO;2-O (2000).

6 Weber-Ban, E. U., Reid, B. G., Miranker, A. D. & Horwich, A. L. Global unfolding of a substrate protein by the Hsp100 chaperone ClpA. *Nature* **401**, 90-93, doi:10.1038/43481 (1999).

7 Defenbaugh, D. A. & Nakai, H. A context-dependent ClpX recognition determinant located at the C terminus of phage Mu repressor. *J Biol Chem* **278**, 52333-52339, doi:10.1074/jbc.M308724200 (2003).

8 Martinez, V. *et al.* CRISPR/Cas9-based genome editing for simultaneous interference with gene expression and protein stability. *Nucleic Acids Res* **45**, e171, doi:10.1093/nar/gkx797 (2017).

9 Takpho, N., Watanabe, D. & Takagi, H. High-level production of valine by expression of the feedback inhibition-insensitive acetohydroxyacid synthase in Saccharomyces cerevisiae. *Metab Eng* **46**, 60-67, doi:10.1016/j.ymben.2018.02.011 (2018).

10 Ofuonye, E., Kutin, K. & Stuart, D. T. Engineering Saccharomyces cerevisiae fermentative pathways for the production of isobutanol. *Biofuels* **4**, 185-201, doi:10.4155/bfs.12.85 (2013).

11 Kopecky, J., Janata, J., Pospisil, S., Felsberg, J. & Spizek, J. Mutations in two distinct regions of acetolactate synthase regulatory subunit from Streptomyces cinnamonensis result in the lack of sensitivity to end-product inhibition. *Biochem Biophys Res Commun* **266**, 162-166, doi:10.1006/bbrc.1999.1792 (1999).

12 Kaplun, A. *et al.* Structure of the regulatory subunit of acetohydroxyacid synthase isozyme III from Escherichia coli. *J Mol Biol* **357**, 951-963, doi:10.1016/j.jmb.2005.12.077 (2006).

13 Hammer, S. K. & Avalos, J. L. Uncovering the role of branched-chain amino acid transaminases in Saccharomyces cerevisiae isobutanol biosynthesis. *Metab. Eng.* **44**, 302-312, doi:https://doi.org/10.1016/j.ymben.2017.10.001 (2017).

14 Rahman, M. M. *et al.* The Crystal Structure of a Bacterial l-Arabinonate Dehydratase Contains a [2Fe-2S] Cluster. *ACS Chem Biol* **12**, 1919-1927, doi:10.1021/acschembio.7b00304 (2017).

15 Entian, K.-D. & Kötter, P. 25 yeast genetic strain and plasmid collections. *Methods in microbiology* **36**, 629-666 (2007).

16 Sikorski, R. S. & Hieter, P. A system of shuttle vectors and yeast host strains designed for efficient manipulation of DNA in Saccharomyces cerevisiae. *Genetics* **122**, 19 (1989).

17 Christianson, T. W., Sikorski, R. S., Dante, M., Shero, J. H. & Hieter, P. Multifunctional yeast high-copy-number shuttle vectors. *Gene* **110**, 119-122 (1992).

18 Zhao, E. M. *et al.* Optogenetic regulation of engineered cellular metabolism for microbial chemical production. *Nature* **555**, 683-687, doi:10.1038/nature26141 (2018).

19 Zhang, Y. *et al.* Xylose utilization stimulates mitochondrial production of isobutanol and 2-methyl-1-butanol in Saccharomyces cerevisiae. *Biotechnol Biofuels* **12**, 223, doi:10.1186/s13068-019-1560-2 (2019).

20 Zhao, E. M. *et al.* Design, characterization, and modeling of rapid optogenetic inverter circuits for dynamic control in yeast metabolic engineering, Under review. (2020).

21 Goldstein, A. L. & McCusker, J. H. Three new dominant drug resistance cassettes for gene disruption in Saccharomyces cerevisiae. *Yeast* **15**, 1541-1553, doi:10.1002/(SICI)1097-0061(199910)15:14<1541::AID-YEA476>3.0.CO;2-K (1999).

22 Avalos, J. L., Fink, G. R. & Stephanopoulos, G. Compartmentalization of metabolic pathways in yeast mitochondria improves the production of branched-chain alcohols. *Nat Biotechnol* **31**, 335-341, doi:10.1038/nbt.2509 (2013).

23 Gueldener, U., Heinisch, J., Koehler, G. J., Voss, D. & Hegemann, J. H. A second set of loxP marker cassettes for Cre-mediated multiple gene knockouts in budding yeast. *Nucleic Acids Res* **30**, e23, doi:10.1093/nar/30.6.e23 (2002).

24 Atsumi, S., Li, Z. & Liao, J. C. Acetolactate synthase from Bacillus subtilis serves as a 2-ketoisovalerate decarboxylase for isobutanol biosynthesis in Escherichia coli. *Appl Environ Microbiol* **75**, 6306-6311, doi:10.1128/AEM.01160-09 (2009).

25 Brinkmann-Chen, S. *et al.* General approach to reversing ketol-acid reductoisomerase cofactor dependence from NADPH to NADH. *Proc Natl Acad Sci U S A* **110**, 10946-10951, doi:10.1073/pnas.1306073110 (2013).

26 Urano, J. E., CO, US), Dundon, Catherine Asleson (Englewood, CO, US). Cytosolic isobutanol pathway localization for the production of isobutanol. United States patent (2012).

27 Batrakou, D. G., Heron, E. D. & Nieduszynski, C. A. Rapid high-resolution measurement of DNA replication timing by droplet digital PCR. *Nucleic Acids Res* **46**, e112, doi:10.1093/nar/gky590 (2018).

28 Kohlhaw, G. B. Leucine Biosynthesis in Fungi: Entering Metabolism through the Back Door. *Microbiol Mol Biol R* **67**, 1-15, doi:10.1128/mmbr.67.1.1-15.2003 (2003).

29 Hazelwood, L. A., Daran, J. M., van Maris, A. J. A., Pronk, J. T. & Dickinson, J. R. The ehrlich pathway for fusel alcohol production: a century of research on Saccharomyces cerevisiae metabolism. *Appl Environ Microb* **74**, 2259-2266, doi:10.1128/Aem.02625-07 (2008).
