# Supplementary material for: Biosensor for branched-chain amino acid metabolism in yeast and applications in isobutanol and isopentanol production
Source: Nat Commun. 2022 Jan 12;13:270. doi: 10.1038/s41467-021-27852-x (PMC8755756; doi:10.1038/s41467-021-27852-x)
Supplement: Supplementary file 2 — Reporting Summary [file 41467_2021_27852_MOESM2_ESM.pdf]

nature research

Corresponding author(s): Jose L. Avalos

Last updated by author(s): Dec 8, 2021

Reporting Summary

Nature Research wishes to improve the reproducibility of the work that we publish. This form provides structure for consistency and transparency in reporting. For further information on Nature Research policies, see our Editorial Policies and the Editorial Policy Checklist.

Statistics

For all statistical analyses, confirm that the following items are present in the figure legend, table legend, main text, or Methods section.

n/a	Confirmed	
☐	☒	The exact sample size (*n*) for each experimental group/condition, given as a discrete number and unit of measurement
☐	☒	A statement on whether measurements were taken from distinct samples or whether the same sample was measured repeatedly
☐	☒	The statistical test(s) used AND whether they are one- or two-sided<br>*Only common tests should be described solely by name; describe more complex techniques in the Methods section.*
☒	☐	A description of all covariates tested
☐	☒	A description of any assumptions or corrections, such as tests of normality and adjustment for multiple comparisons
☐	☒	A full description of the statistical parameters including central tendency (e.g. means) or other basic estimates (e.g. regression coefficient) AND variation (e.g. standard deviation) or associated estimates of uncertainty (e.g. confidence intervals)
☐	☒	For null hypothesis testing, the test statistic (e.g. *F*, *t*, *r*) with confidence intervals, effect sizes, degrees of freedom and *P* value noted<br>*Give P values as exact values whenever suitable.*
☒	☐	For Bayesian analysis, information on the choice of priors and Markov chain Monte Carlo settings
☐	☒	For hierarchical and complex designs, identification of the appropriate level for tests and full reporting of outcomes
☒	☐	Estimates of effect sizes (e.g. Cohen's *d*, Pearson's *r*), indicating how they were calculated

Our web collection on statistics for biologists contains articles on many of the points above.

Software and code

Policy information about availability of computer code

Data collection	Agilent Chemstation, FACSDiva (V.8.0.2), and FlowJo
Data analysis	Microsoft Excel, FlowJo, Graphpad Prism

For manuscripts utilizing custom algorithms or software that are central to the research but not yet described in published literature, software must be made available to editors and reviewers. We strongly encourage code deposition in a community repository (e.g. GitHub). See the Nature Research guidelines for submitting code & software for further information.

Data

Policy information about availability of data

All manuscripts must include a data availability statement. This statement should provide the following information, where applicable:

- Accession codes, unique identifiers, or web links for publicly available datasets
- A list of figures that have associated raw data
- A description of any restrictions on data availability

The authors declare that all data supporting the findings of this study are available within the paper (and its Supplementary Information files). The source data underlying Figs. 1-6, Supplementary Figs. S2-7, S9, S10, S12, S13, S15, S16, and Supplementary Table 3 are provided in the Source Data File.

Field-specific reporting

Life sciences study design

All studies must disclose on these points even when the disclosure is negative.

Sample size	Sample sizes were n >= 3 so that p-values calculated from a standard t-test would yield a one-tail probability of lower than 0.05. this is common practice in the fields of biosensor (e.g. Skjoedt et al. Nature Chemical Biology 2016) and metabolic engineering (e.g. Santos et al. Proc Nat Acad Sci 2012, Liu et al. Nat Comms 2019).
Data exclusions	The authors did not exclude any data.
Replication	All experimental findings are reliably reproduced. We have reproduced each experiment at least twice to ensure reliability.
Randomization	All samples in the same experimental group were biological replicates (same genetic makeup but different original colony). We did not need to randomize any experiments because all statistics were done with genetically identical samples.
Blinding	Investigators were blind to the group allocation during data acquisition and analysis.

Reporting for specific materials, systems and methods

We require information from authors about some types of materials, experimental systems and methods used in many studies. Here, indicate whether each material, system or method listed is relevant to your study. If you are not sure if a list item applies to your research, read the appropriate section before selecting a response.

Materials & experimental systems

n/a	Involved in the study	
☒	☐	Antibodies
☒	☐	Eukaryotic cell lines
☒	☐	Palaeontology and archaeology
☒	☐	Animals and other organisms
☒	☐	Human research participants
☒	☐	Clinical data
☒	☐	Dual use research of concern

Methods

n/a	Involved in the study	
☒	☐	ChIP-seq
☐	☒	Flow cytometry
☒	☐	MRI-based neuroimaging

Flow Cytometry

Plots

Confirm that:

☒ The axis labels state the marker and fluorochrome used (e.g. CD4-FITC).

☒ The axis scales are clearly visible. Include numbers along axes only for bottom left plot of group (a 'group' is an analysis of identical markers).

☒ All plots are contour plots with outliers or pseudocolor plots.

☒ A numerical value for number of cells or percentage (with statistics) is provided.

Methodology

Sample preparation	Fluorescence measurements and FACS were performed on samples in mid-exponential growth phase. For all strains, cultures used for flow cytometry assays were diluted to an OD600 of approximately 0.1 with PBS. Samples used for FACS were diluted in fresh medium identical to their growth medium to OD600 of approximately 0.8.
Instrument	A BD LSRII Multi-Laser flow cytometer equipped with FACSDiva software V.8.0.2 (BD Biosciences, San Jose, CA) was used to quantify yEGFP fluorescence at an excitation wavelength of 488 nm and an emission wavelength of 510 nm (525/50 nm bandpass filter). A BD FACSAria Fusion flow cytometer with FACSDiva software was used for fluorescence activated cell sorting (FACS) with a 488 nm excitation wavelength and a bandpass filter of 530/30 for yEGFP detection.
Software	FACSDiva software V.8.0.2 and FlowJo X software (BD Biosciences, San Jose, CA) were used to collect and analysis flow cytometry data.
Cell population abundance	The typical sample size was 50,000 events per measurement of the median fluorescence intensity (MFI).
Gating strategy	Cells were gated on forward scatter (FSC) and side scatter (SSC) signals to discard debris and probable cell aggregates.

☒ Tick this box to confirm that a figure exemplifying the gating strategy is provided in the Supplementary Information.
